# Author Correction: Benchmarking of cell type deconvolution pipelines for transcriptomics data

**DOI:** 10.1038/s41467-020-20288-9

**Published:** 2020-12-02

**Authors:** Francisco Avila Cobos, José Alquicira-Hernandez, Joseph E. Powell, Pieter Mestdagh, Katleen De Preter

**Affiliations:** 1 Center for Medical Genetics Ghent, Department of Biomolecular Medicine, Ghent University, Ghent, Belgium; 2 Cancer Research Institute Ghent (CRIG), Ghent, Belgium; 3 Garvan Weizmann Centre for Cellular Genomics, Garvan Institute of Medical Research, Sydney, NSW Australia; 4 Institute for Molecular Bioscience, University of Queensland, Brisbane, QLD Australia

**Keywords:** Computational biology and bioinformatics, Transcriptomics

Correction to: *Nature Communications* 10.1038/s41467-020-19015-1, published online 6 November 2020.

The original version of this Article contained an error in the Acknowledgements:

‘This work was supported by the European Union’s Horizon 2020 research and innovation programme under grant agreement 668858’.

The number 668858 should have read 826121.

This has now been corrected in both the PDF and HTML versions of the Article.

