# Supplementary material for: Benchmarking of cell type deconvolution pipelines for transcriptomics data
Source: Nat Commun. 2020 Nov 6;11:5650. doi: 10.1038/s41467-020-19015-1 (PMC7648640; doi:10.1038/s41467-020-19015-1)
Supplement: Supplementary file 1 — Supplementary Information [file 41467_2020_19015_MOESM1_ESM.pdf]

SUPPLEMENTARY INFORMATION

Benchmarking of cell type deconvolution pipelines for transcriptomics data

Avila Cobos et al.

Supplementary Notes

Approximation of bulk transcriptomes as linear mixtures

We downloaded publicly available raw poly(A) RNA-sequencing data (Illumina HiSeq 2000; paired-end) from the Sequence Read Archive (SRA; NCBI) of two bulk PBMCs from healthy donors and constituent (bulk) B cells, Monocytes, Natural Killer and T cells together with the corresponding cell type proportions measured by flow cytometry (Supplementary File 3C from https://elifesciences.org/articles/26476/figures#supp3):

SRA accession	Cell type	Donor
SRR1740034	B cells	HD30
SRR1740038	Myeloid DC	HD30
SRR1740042	Monocytes	HD30
SRR1740046	Neutrophils	HD30
SRR1740050	NK cells	HD30
SRR1740054	PBMC	HD30
SRR1740058	T cells	HD30
SRR1740062	B cells	HD31
SRR1740066	Myeloid DC	HD31
SRR1740070	Monocytes	HD31
SRR1740074	Neutrophils	HD31
SRR1740078	NK cells	HD31
SRR1740082	PBMC	HD31
SRR1740086	T cells	HD31

Supplementary Table 1 – Sequence Read Archive (SRA) accession IDs of the different bulk poly(A) RNA-sequencing samples (cell types) used.

We aligned the RNA-seq reads against the human genome (Homo sapiens; Ensembl v91; GRCh38) with STAR v2.6.0c and the output .bam files from the previous step were used as input for HTSeq v0.11.0, resulting in a final matrix with gene counts.

Since we have the three components for the problem T = C · P (see section “Computational deconvolution: formulation and methodologies”; T made of “PBMCs”; C composed of “B cells, Monocytes, Myeloid DCs, Neutrophils, NK and T cells”; P from flow cytometry), we used R^2 (proportion of the variance in the dependent variable that is predictable from the independent variable(s) and also indicates the goodness of fit of the model) as proxy to demonstrate that real bulk transcriptomes can be approximated as linear mixtures of the constituent cell types (=explanatory factors).

The output was R^2_{HD30} = 0.962 and R^2_{HD31} = 0.942, meaning that >94% of the variance in real bulk PBMC transcriptomes is explained by choosing a linear mixing of its constituent CTs and therefore, demonstrating that bulk transcriptomes can therefore be approximated as such linear mixtures.

Small impact of cell cycle in the deconvolution results

The impact of cell cycle phases has been largely overlooked in the deconvolution field in general and current deconvolution frameworks assume cell-type specific markers to be insensitive or invariant to this factor. To investigate the impact of the cell cycle on deconvolution, we used the Baron et al. and PBMC single-cell RNA-seq datasets as input for the "cyclone" function developed by Scialdone *et al.*[1] as part of the "scran" package (R statistical programming language), which contains a pre-trained set of human marker gene pairs that allows the classification of cells into different cell cycle phases.

After cells were initially classified into G1, S and G2M, we binarized the cells into "S" (proliferating) and "non-S" (= composed of G1 and G2M; non-proliferating) and, for each of the ratios 0:100 / 25:75 / 50:50 / 75:25 / 100:0 (% of S: % non-S), we artificially generated 100 pseudo-bulk mixtures of 100 cells each to evaluate the deconvolution framework "linear scale + LogNormalize + nnls". The scatter plots below revealed, for both datasets, very small differences in RMSE and Pearson correlation values across the different ratios (Baron: $\Delta_{RMSE} = 0.06 – 0.04 = 0.02$; $\Delta_{Pearson} = 0.96 – 0.93 = 0.03$; PBMCs: $\Delta_{RMSE} = 0.05 – 0.03 = 0.02$; $\Delta_{Pearson} = 1 – 0.96 = 0.04$). Of note, for the PBMC data, most of the cells used in the pseudo-bulk mixtures with 100:0 ratio are T-cells because other cell types were found to have fewer cells in cell cycle stage "S".

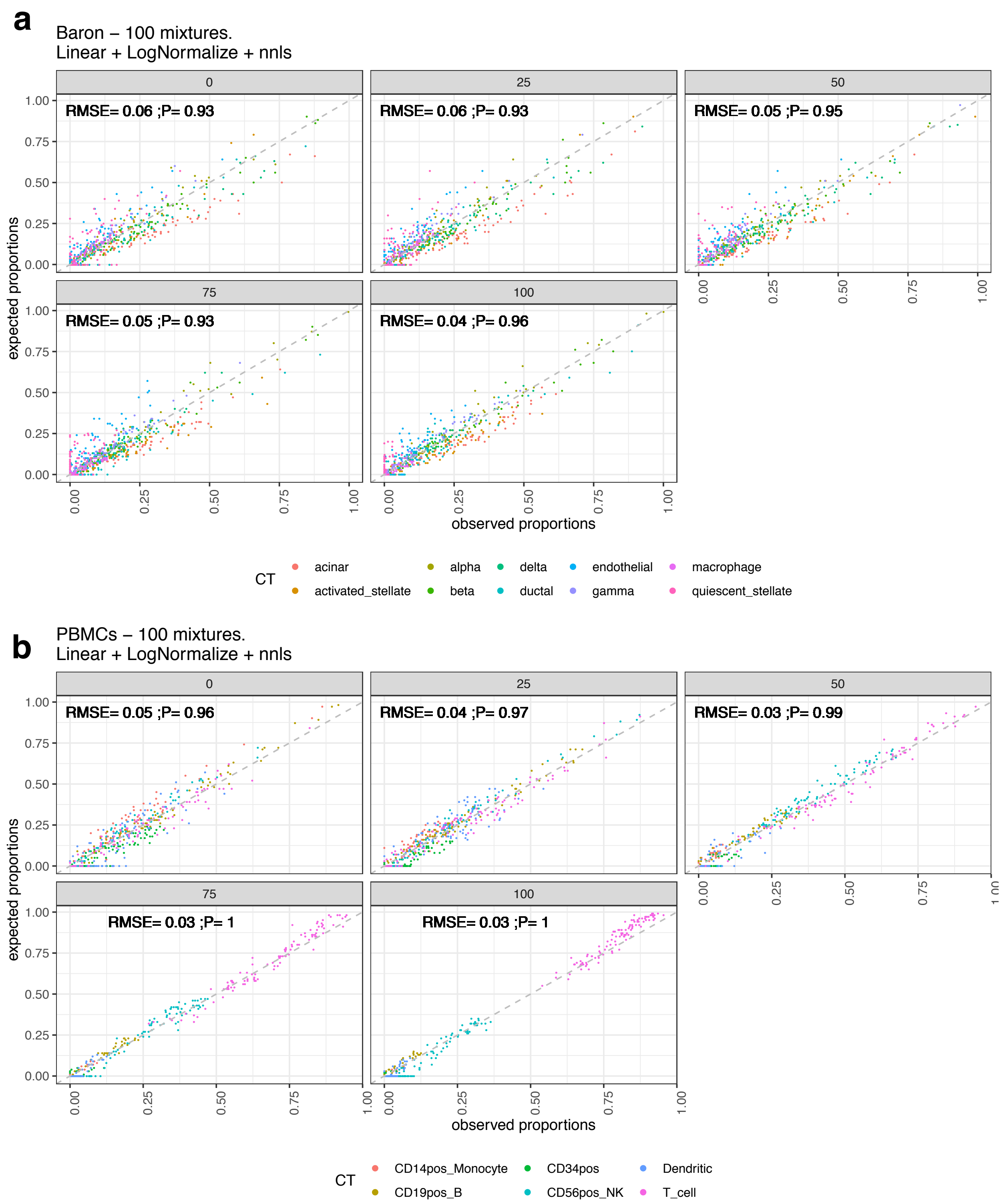

Supplementary Figure 1 – Small impact of cell cycle stage in the deconvolution results on a) Baron; b) PBMCs datasets, respectively. The number in each gray rectangle depicts the percentage of cells in S phase and, for each percentage, 100 pseudo-bulk mixtures were assessed with nnls (data in linear scale followed by LogNormalize).

Fold change evaluation of cell-type-specific markers used with bulk deconvolution methodologies

For all markers across each dataset, we took a closer look at the fold-change distribution for both the cell type where they were initially found as marker (highest fold changes) and the fold-change differences among all other cell types. Using the threshold values used to select a gene as marker, we computed the percentage of those that could also be considered markers for a secondary cell type (values between parentheses in the boxplots below). For the five datasets included in the benchmark, 7 to 38% of the markers were not “specific” (exclusive) for only one cell type.

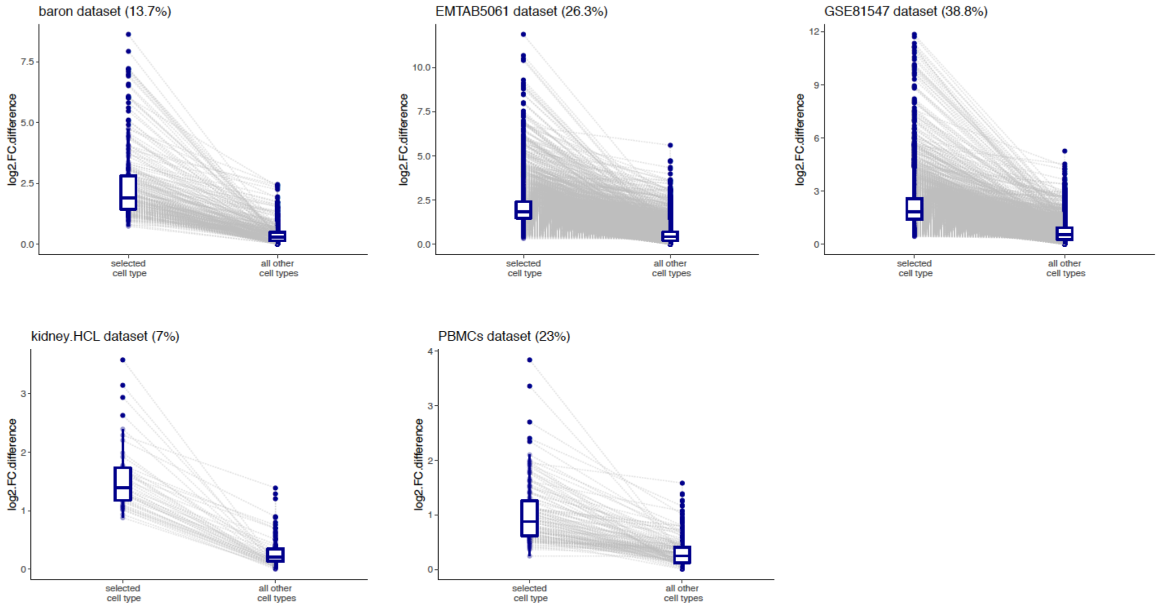

Supplementary Figure 2 – Fold change values for the different cell-type specific markers across the different datasets used in the manuscript. Left boxplots depict the fold change for the cell type where markers were originally found and right boxplots depict the fold changes for the remaining cell types present in the dataset.

Processing poly(A) RNA-seq of nine human bulk PBMCs samples

We downloaded raw poly(A) RNA-seq (single-end) data for the following samples:

donor ID	SRA accession
donor_1	SRR6337113
donor_10	SRR6337120
donor_12	SRR6337121
donor_2	SRR6337114
donor_4	SRR6337115
donor_5	SRR6337116
donor_6	SRR6337117
donor_7	SRR6337118
donor_9	SRR6337119

Supplementary Table 2 – Sequence Read Archive (SRA) accession IDs of the nine bulk poly(A) RNA-sequencing samples (PBMCs).

And we aligned the RNA-seq reads against the human genome (Homo sapiens; Ensembl v91; GRCh38) with STAR v2.6.0c and the output .bam files from the previous step were used as input for HTSeq v0.11.0, resulting in a final matrix with gene counts.

Incompatible data transformations or normalizations with several deconvolution methods

Global and column z-score normalizations generated negative values, making it incompatible with the deconvolution methods that use single-cell RNA-seq data as reference and with bulk deconvolution methods such as DeconRNASeq, ssKL, ssFrob and DSA. Quantile normalization is used by default in FARDEEP but we disabled it to observe the impact of other scaling/normalization strategies. Row scaling led to singular matrices (several rows were identical; determinant = 0) and thus methods such as robust linear regression (RLR) failed. Linnorm normalization performs an internal logarithmic transformation step, so it is not compatible with logarithmic, square-root and VST transformed input data. CIBERSORT performs and internal z-score standardization of the input matrices prior to fit the support vector regression. The glmnet function used in penalized regression approaches such as ridge, lasso, elastic net and DCQ, includes an internal standardization step (=predictors to be scaled as z-scores) to ensure that the penalty affects each coefficient equally. DSA, ssFrobenius and ssKL can only be applied to data in linear scale [http://web.cbio.uct.ac.za/~renaud/CRAN/web/CellMix/gedAlgorithm.ssKL.html] whereas dtangle only accepts input matrices in logarithmic scale. ssFrobenius performs an internal mean-centering step of each signature separately whereas in ssKL no re-scaling is performed at all. VST and median ratios normalization were not possible with the kidney.HCL scRNA-seq dataset because every gene contained at least one zero, hampering the computation of log geometric means and the Linnorm normalization was unfeasible in the PBMCs dataset because it required at least 200 genes where 75% of the samples had non-zero values. MuSiC and SCDC could not be tested using PBMCs because n=1 (they are "multi-subject" methods). deconvSeq is formulated as a generalized linear model that accounts for the quadratic relationship between the mean and the variance in RNA-seq count data using the log link function for a negative binomial distribution, so it is not compatible with logarithmic, square-root and vst transformed input data, and it requires the input to be un-normalized. DWLS includes an internal log2 transformation step followed by differential gene expression analysis (internal marker selection step) with Model-based Analysis of Single-cell Transcriptomics (MAST)[2]. For these reasons, only single-cell input data in linear scale and normalization strategies not generating negative or bounded values were compatible with DWLS.

Explicit versus implicit non-negativity and sum-to-one constraints

For some methods, the output needed to be explicitly ("E" in the table below) modified after the deconvolution to enforce only positive proportions (non-negativity constraint: negative proportions were set to 0) and that they sum to one. For others, these constraints were implicitly ("I" in the table below) included and the output was left unchanged.

deconvolution method	non-negativity	sum-to-one
OLS	E	E
NNLS	I	E
FARDEEP	I	E
RLR	E	E
lasso	E	E
ridge	E	E
elastic net	E	E
DCQ	E	E
DSA	E	E
EPIC	I	I
dtangle	I	I
DeconRNASeq	I	I
CIBERSORT	I	I*
ssFrobenius	I	I
ssKL	I	I
deconvSeq	I	I
MuSiC	I	I
SCDC	I	I
Bisque	I	I
DWLS	E	E**

Supplementary Table 3 – Explicit (E) versus implicit (I) non-negativity and sum-to-one constraints

(*) Users can select “absolute” mode to remove the sum-to-one constraint or to use a signature score as output. (**) It only included the implicit sum-to-one constraint. Thus, to enforce both constraints, we artificially enforced the non-negativity constraint followed by sum-to-one.

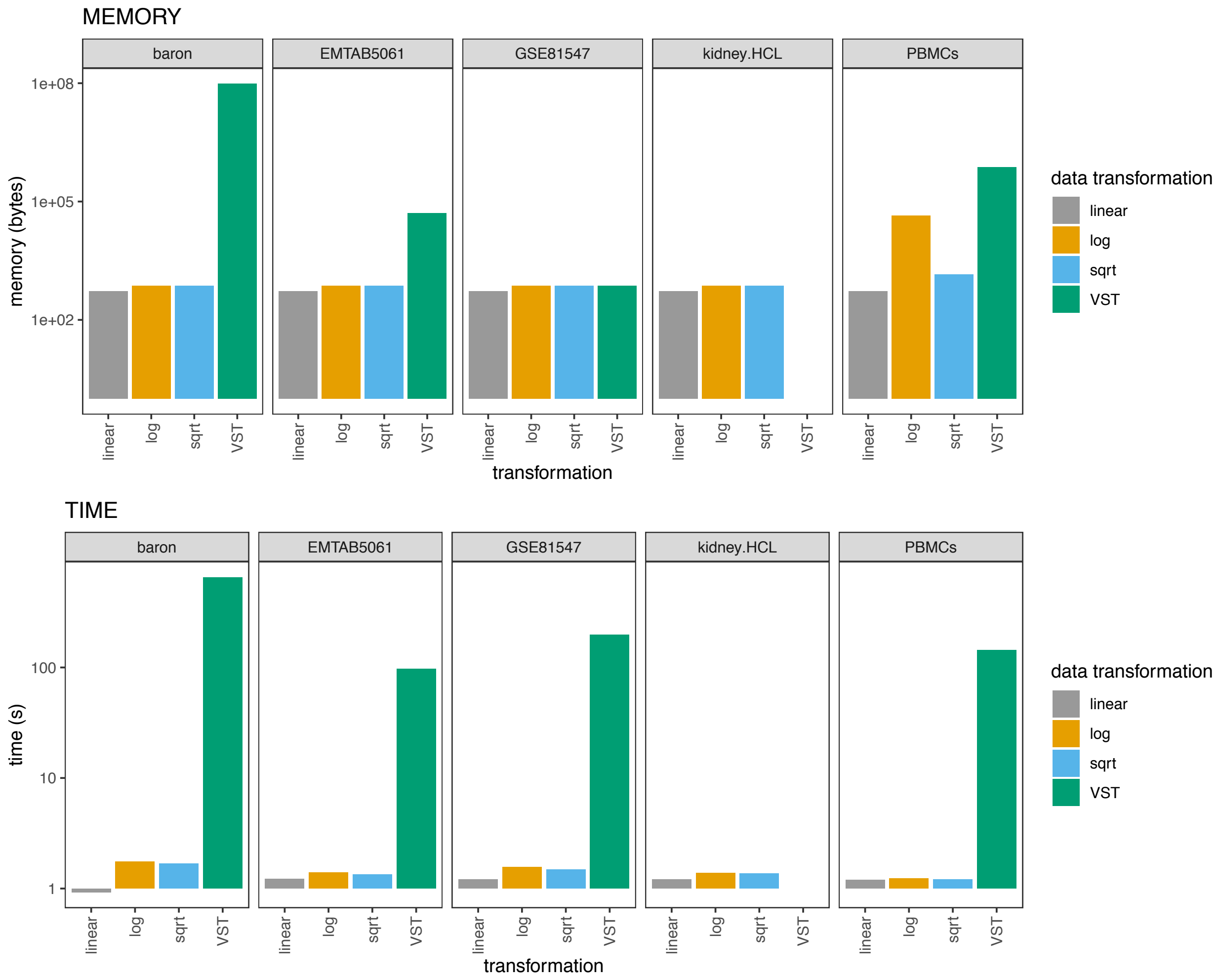

Supplementary Figure 3 – RAM memory (bytes) and time requirements (seconds) for the different transformations across scRNA-seq datasets. “none” represents the data un-transformed, in linear scale; log = logarithmic; sqrt = square-root; VST = variance stabilization transformation. VST was not possible with the kidney.HCL dataset because every gene contained at least one zero, hampering the computation of log geometric means.

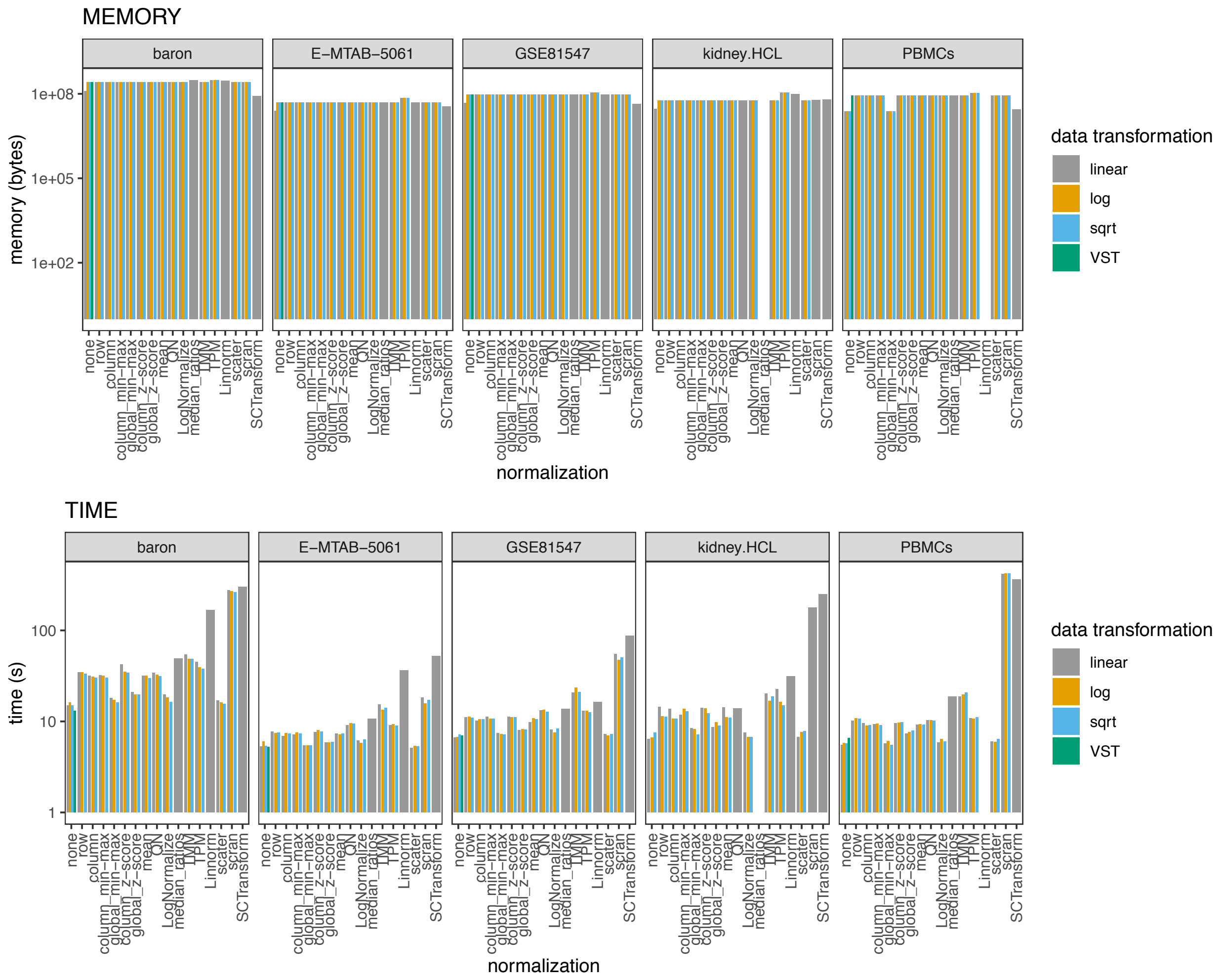

Supplemental Figure 4 – RAM memory (bytes) and time requirements (seconds) for the different scaling/normalization strategies across different single-cell RNA-seq datasets.

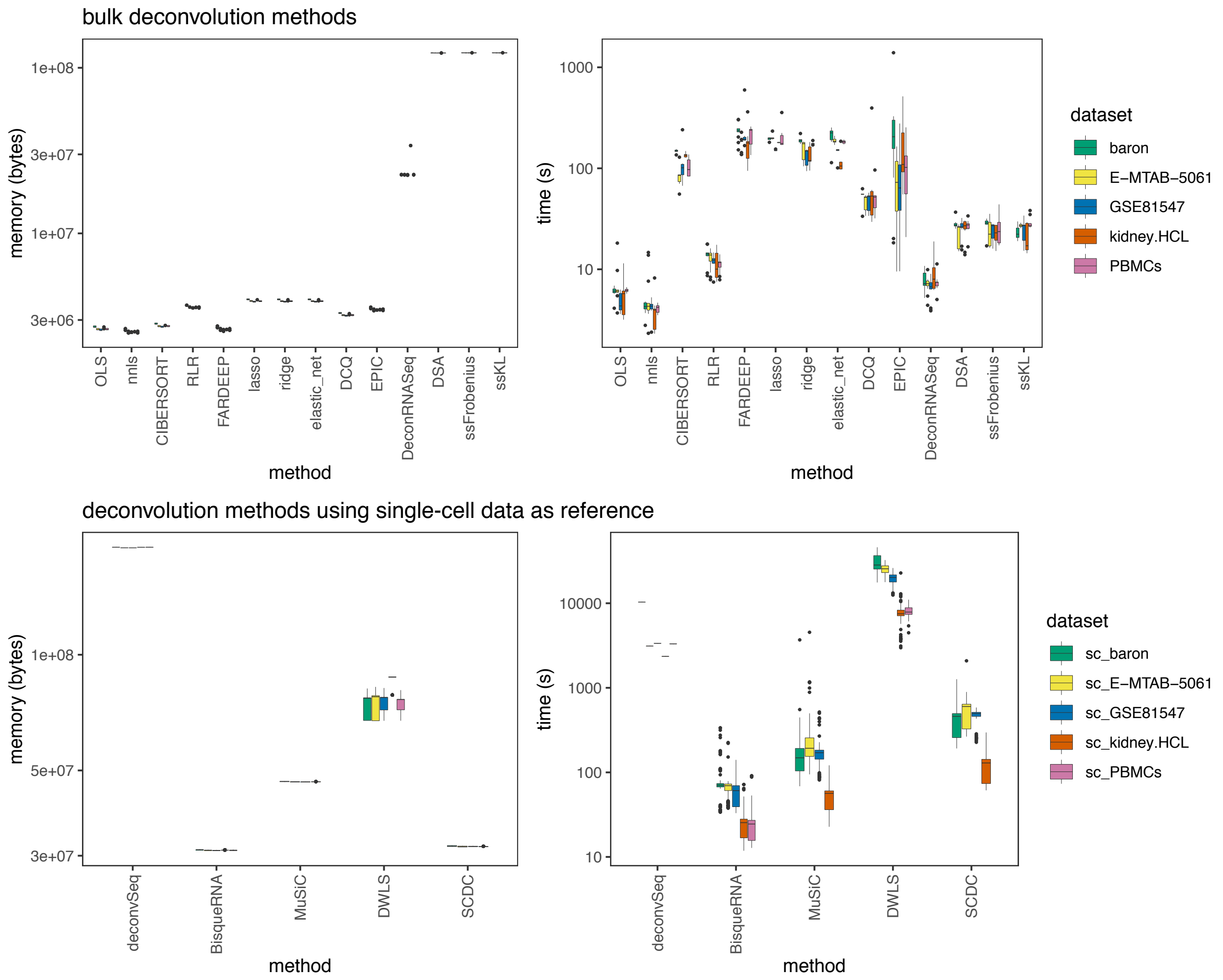

Supplementary Figure 5 - RAM memory (bytes) (left hand side) and time (seconds) (right hand side) requirements for the different bulk deconvolution methodologies (top panel) and deconvolution methods using single-cell RNA-seq data as reference (bottom panel) across datasets with expression values in linear scale (boxplots depict all scaling/normalization strategies (n=16 for bulk; n=20 for single-cell) across all pseudo-bulk cell pool sizes:).

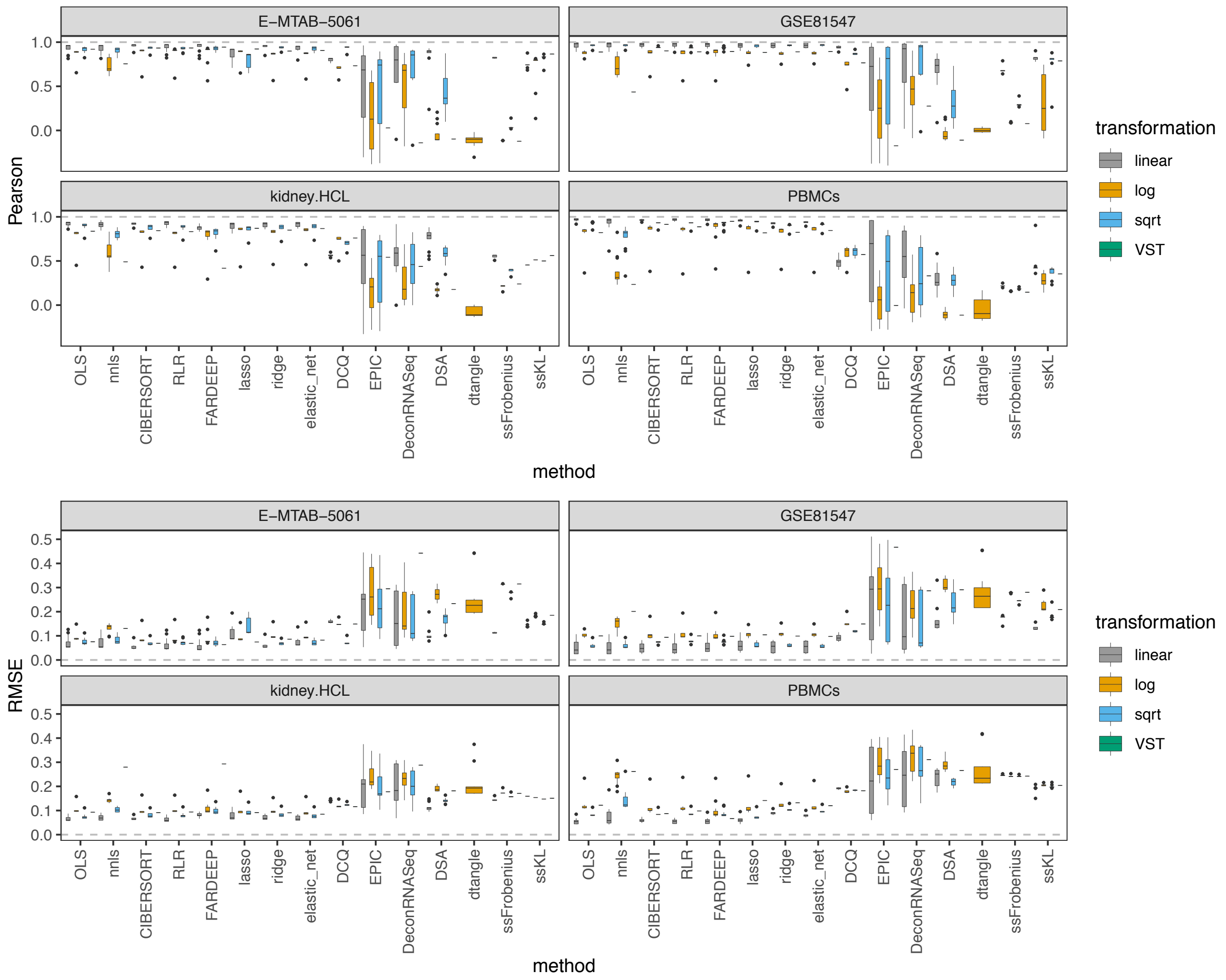

Supplementary Figure 6 – Pearson correlation (top panel) and RMSE values (bottom panel) between the known proportions in 1000 pseudo-bulk tissue mixtures from the E-MTAB-5061, GSE81547, kidney.HCL and PBMCs datasets (pool size = 100 cells per mixture) and the predicted proportions from the different bulk deconvolution methods.

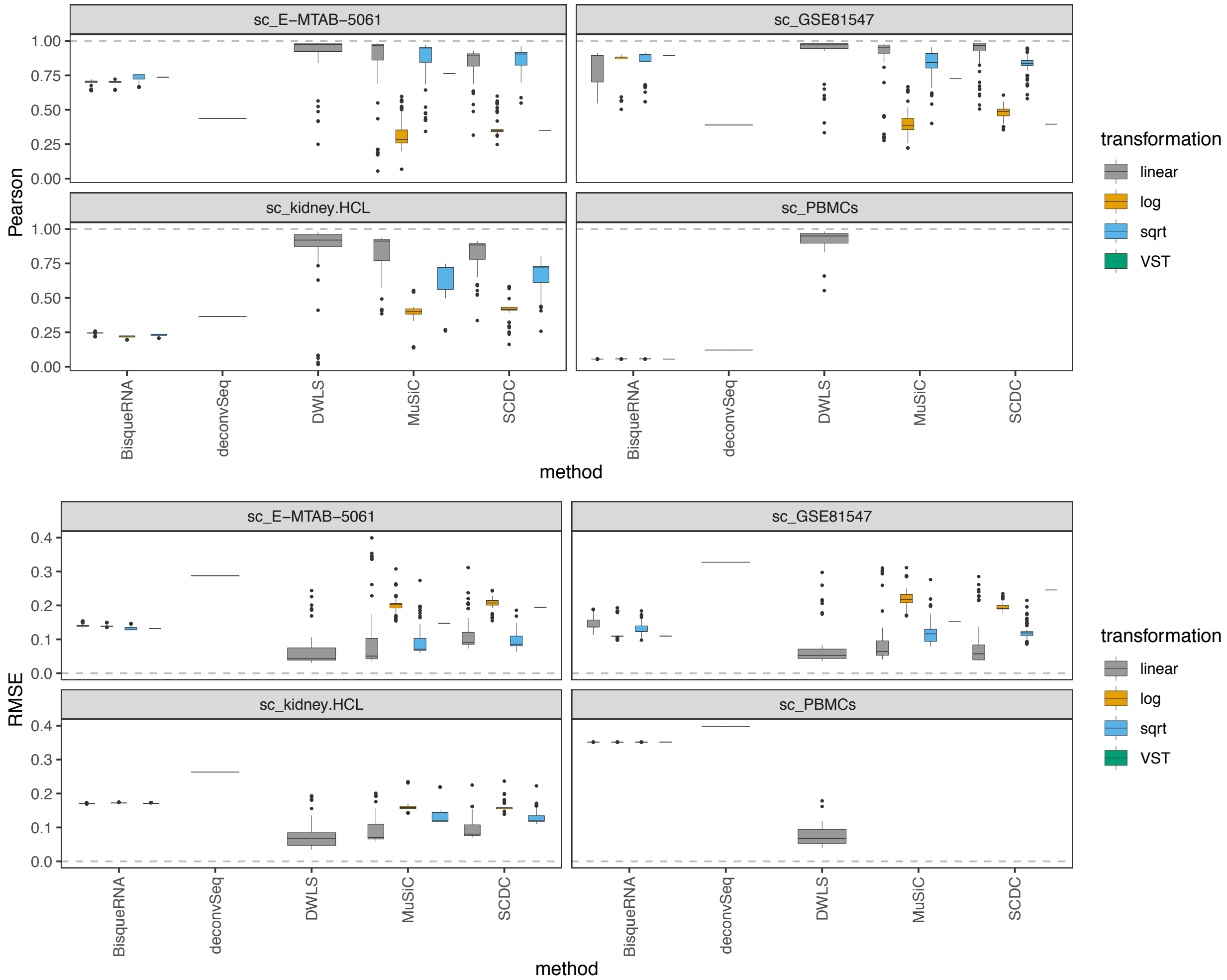

Supplementary Figure 7 – Pearson correlation (top panel) and RMSE values (bottom panel) between the known proportions in 1000 pseudo-bulk tissue mixtures from the E-MTAB-5061, GSE81547, kidney.HCL and PBMCs datasets (pool size = 100 cells per mixture) and the predicted proportions from the different deconvolution methods that use single-cell RNA-seq as reference. MuSiC and SCDC were not applicable to the PBMC dataset because it requires the number of samples to be greater than one. Each boxplot contains all normalization strategies that were tested in combination with a given method.

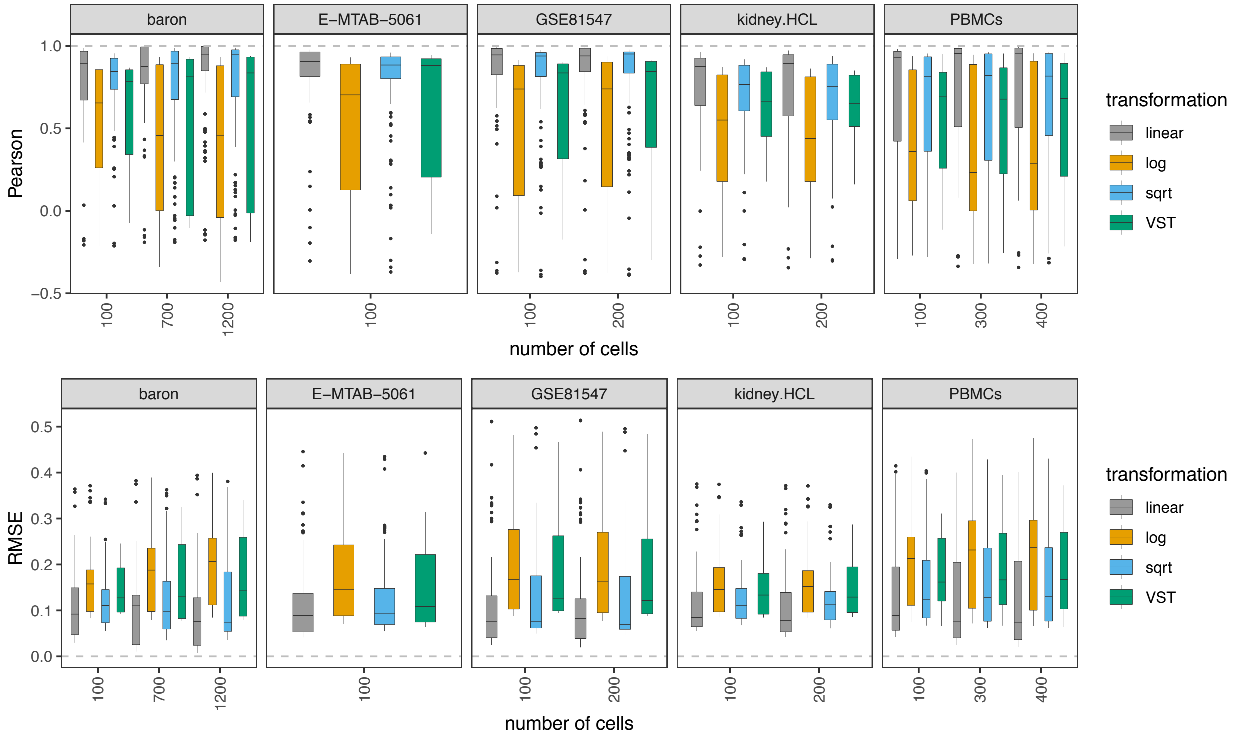

Supplementary Figure 8 – Pearson correlation (top panel) and RMSE values (bottom panel) between the known proportions in 1000 pseudo-bulk tissue mixtures and the predicted proportions from the different bulk deconvolution methods. Each boxplot contains all combinations of method and normalization strategies that were tested with a given cell pool size.

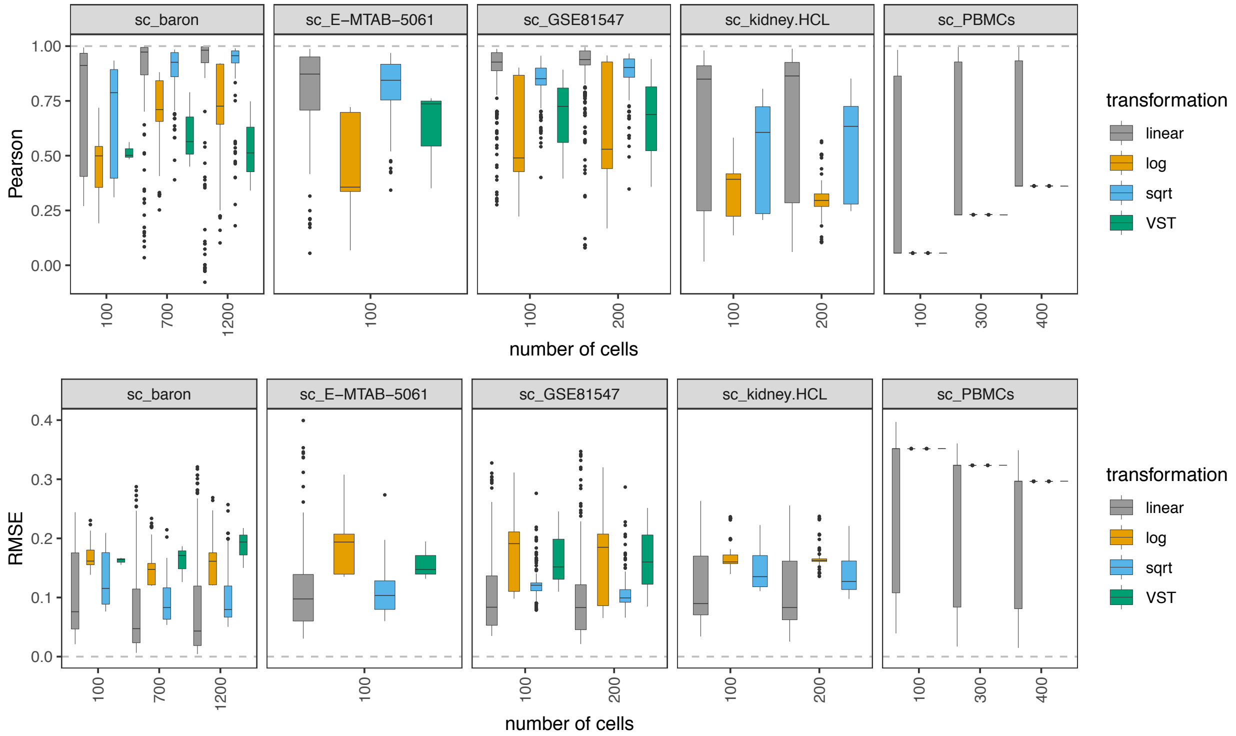

Supplementary Figure 9 – Pearson correlation (top panel) and RMSE values (bottom panel) between the known proportions in 1000 pseudo-bulk tissue mixtures and the predicted proportions from the different deconvolution methods that use single-cell RNA-seq data as reference. Each boxplot contains all combinations of method and normalization strategies that were tested with a given cell pool size.

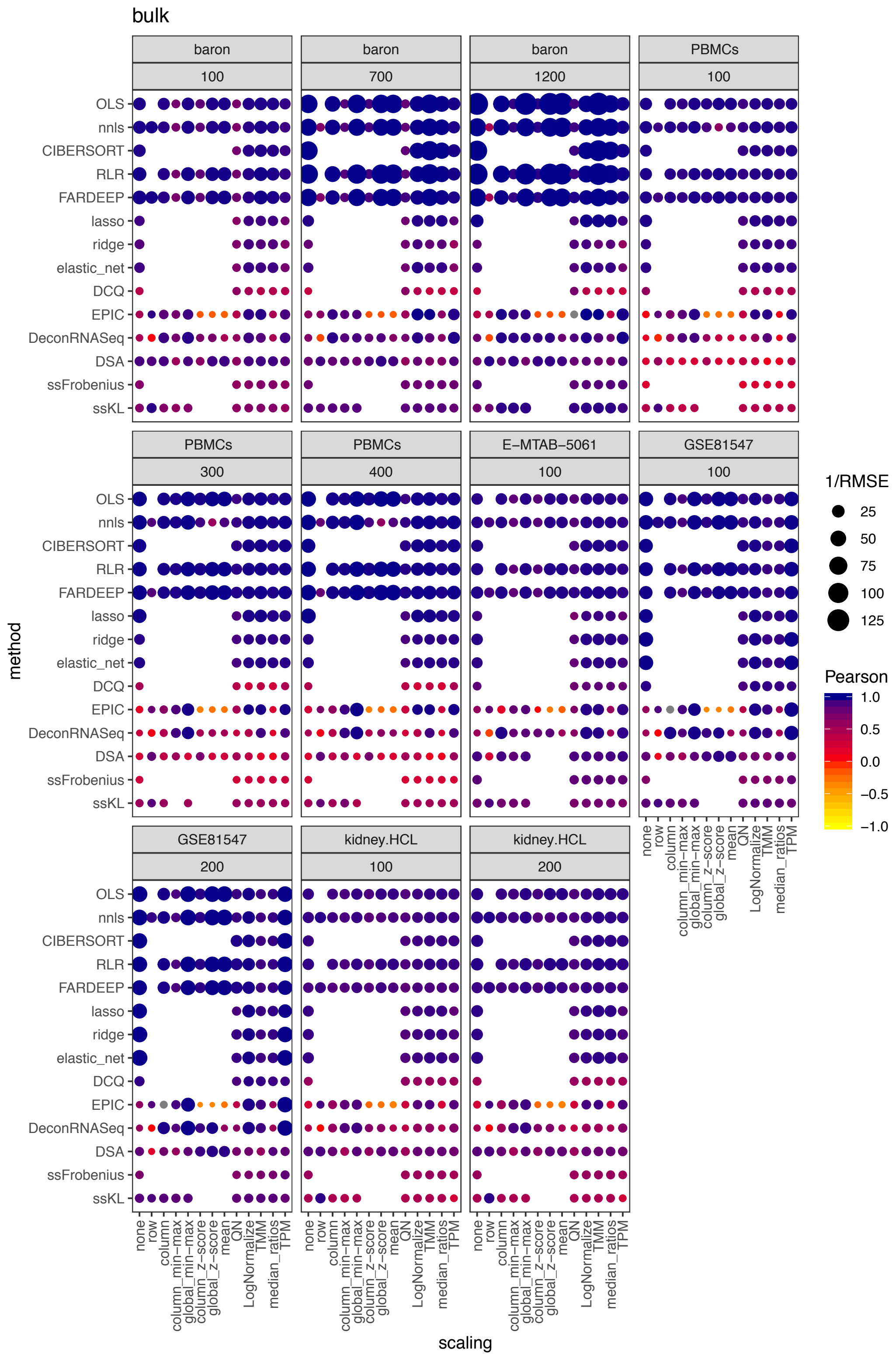

Supplementary Figure 10 – Pearson correlation values between the expected (known) proportions in 1000 pseudo-bulk tissue mixtures in linear scale (several datasets and pool sizes, as depicted in the grey labels) and the output proportions from the different bulk deconvolution methods. The darker the blue and the higher the area of the circle represents higher Pearson and lower RMSE values, respectively.

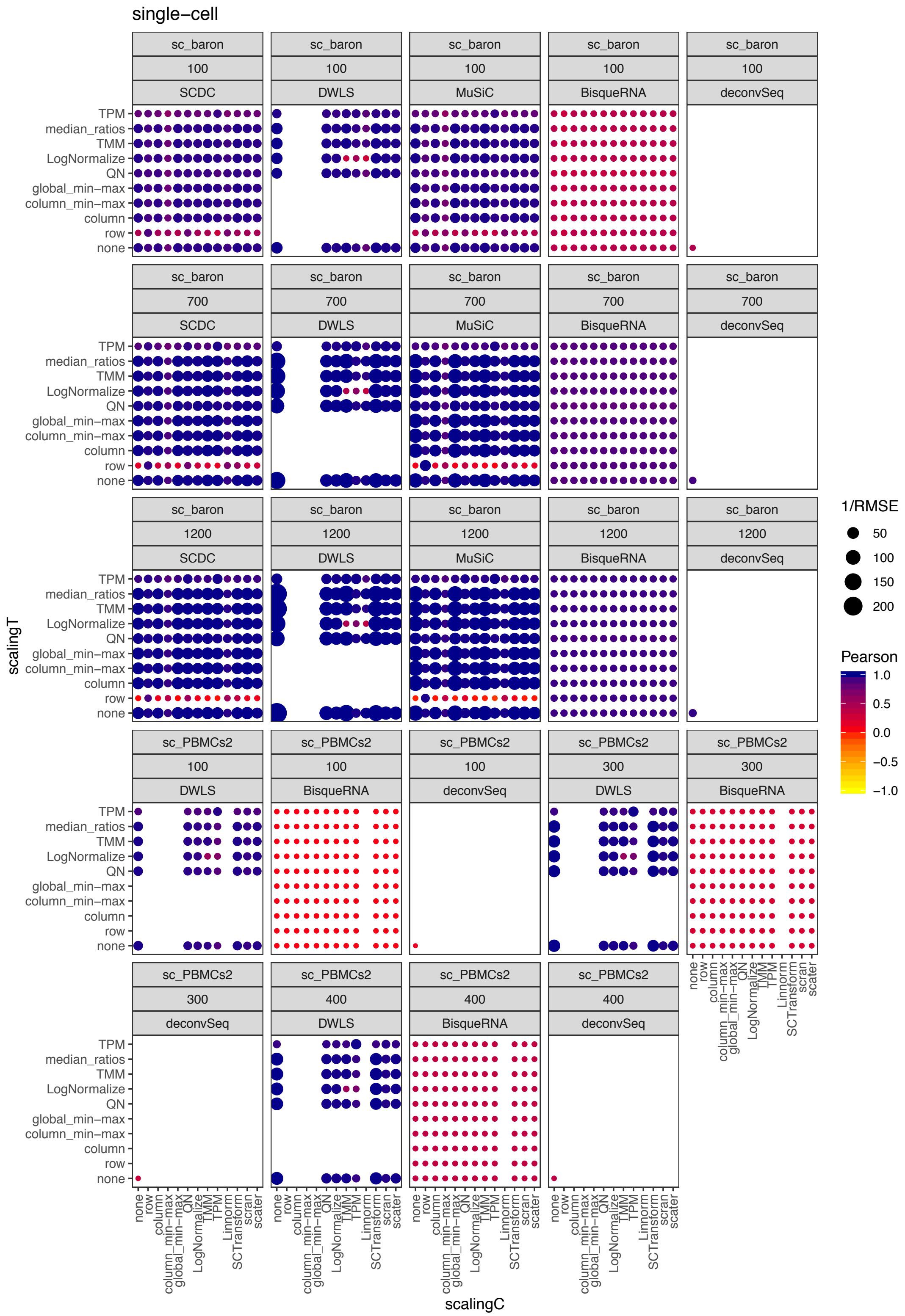

Supplementary Figure 11a – Pearson correlation values between the expected (known) proportions in 1000 pseudo-bulk tissue mixtures in linear scale (several pool sizes and datasets, as depicted in the grey labels) and the output proportions from the different deconvolution methods that use single-cell RNA-seq data as reference. The darker the blue and the higher the area of the circle represents higher Pearson and lower RMSE values, respectively.

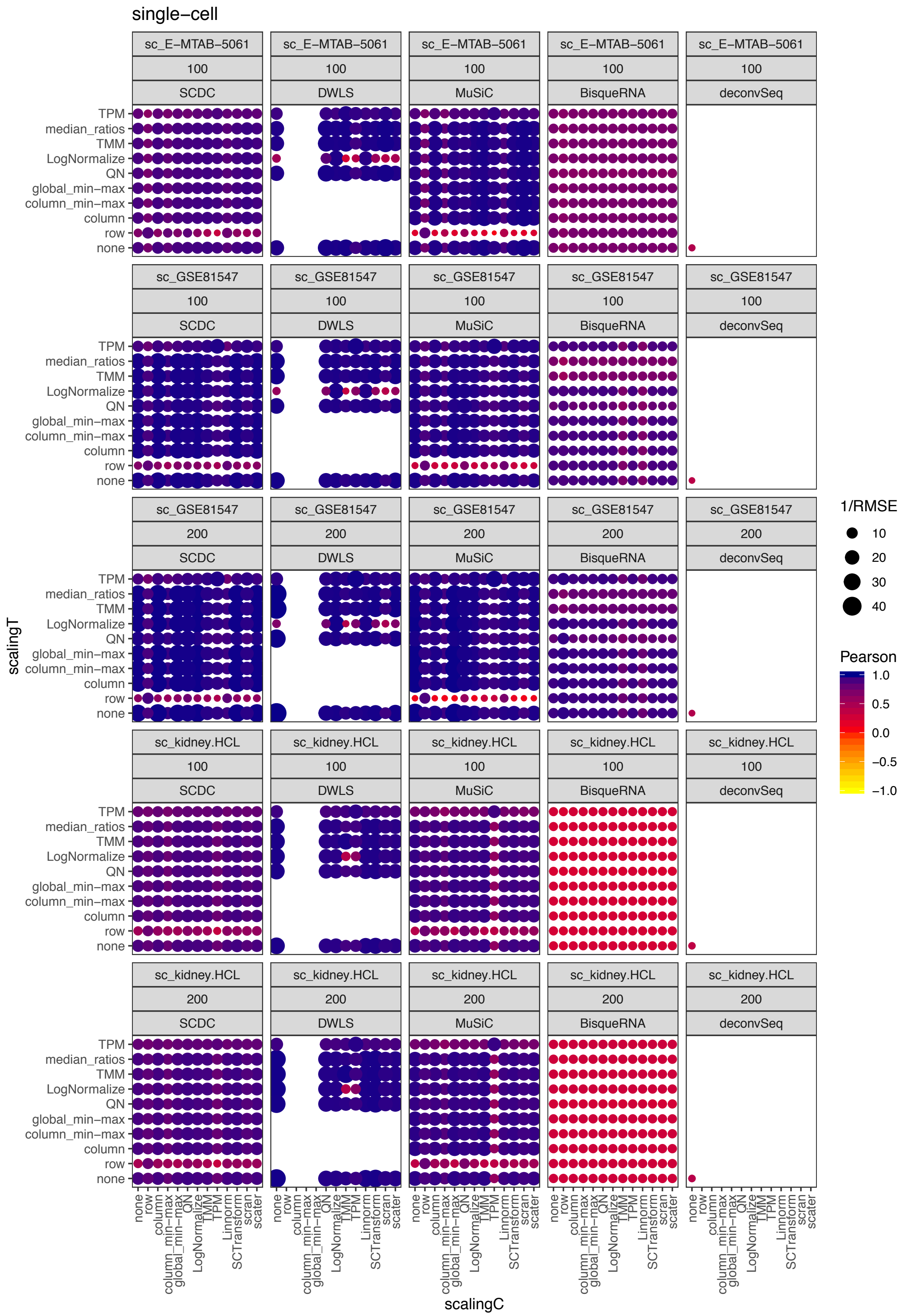

Supplementary Figure 11b – Pearson correlation values between the expected (known) proportions in 1000 pseudo-bulk tissue mixtures in linear scale (several pool sizes and datasets, as depicted in the grey labels) and the output proportions from the different deconvolution methods that use single-cell RNA-seq data as reference. The darker the blue and the higher the area of the circle represents higher Pearson and lower RMSE values, respectively.

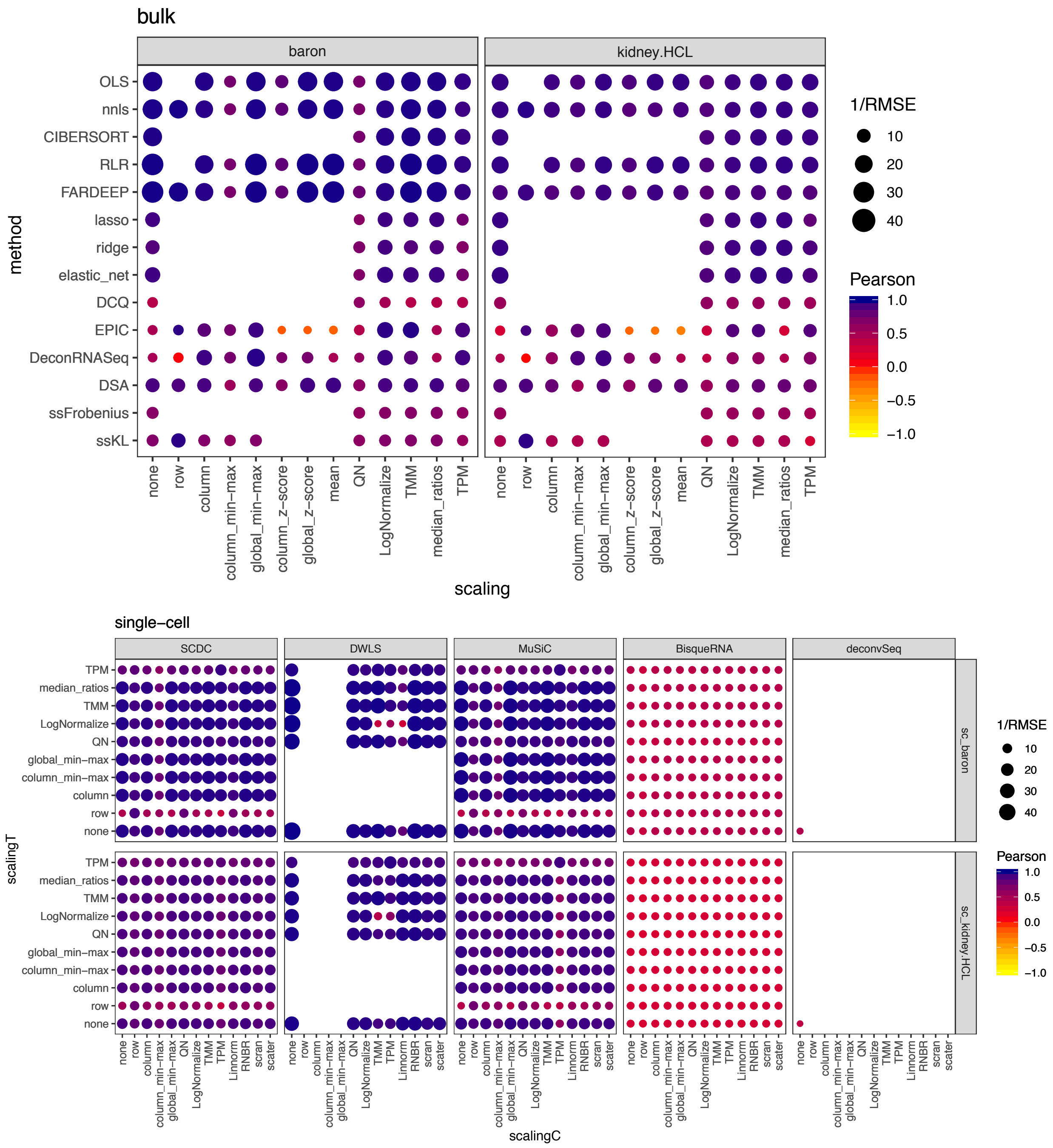

Supplementary Figure 12 – Similar Pearson correlation and RMSE values between pancreas (baron dataset) and kidney.HCL datasets for both bulk deconvolution methods (top panel) and those that use scRNA-seq as reference (bottom panel).

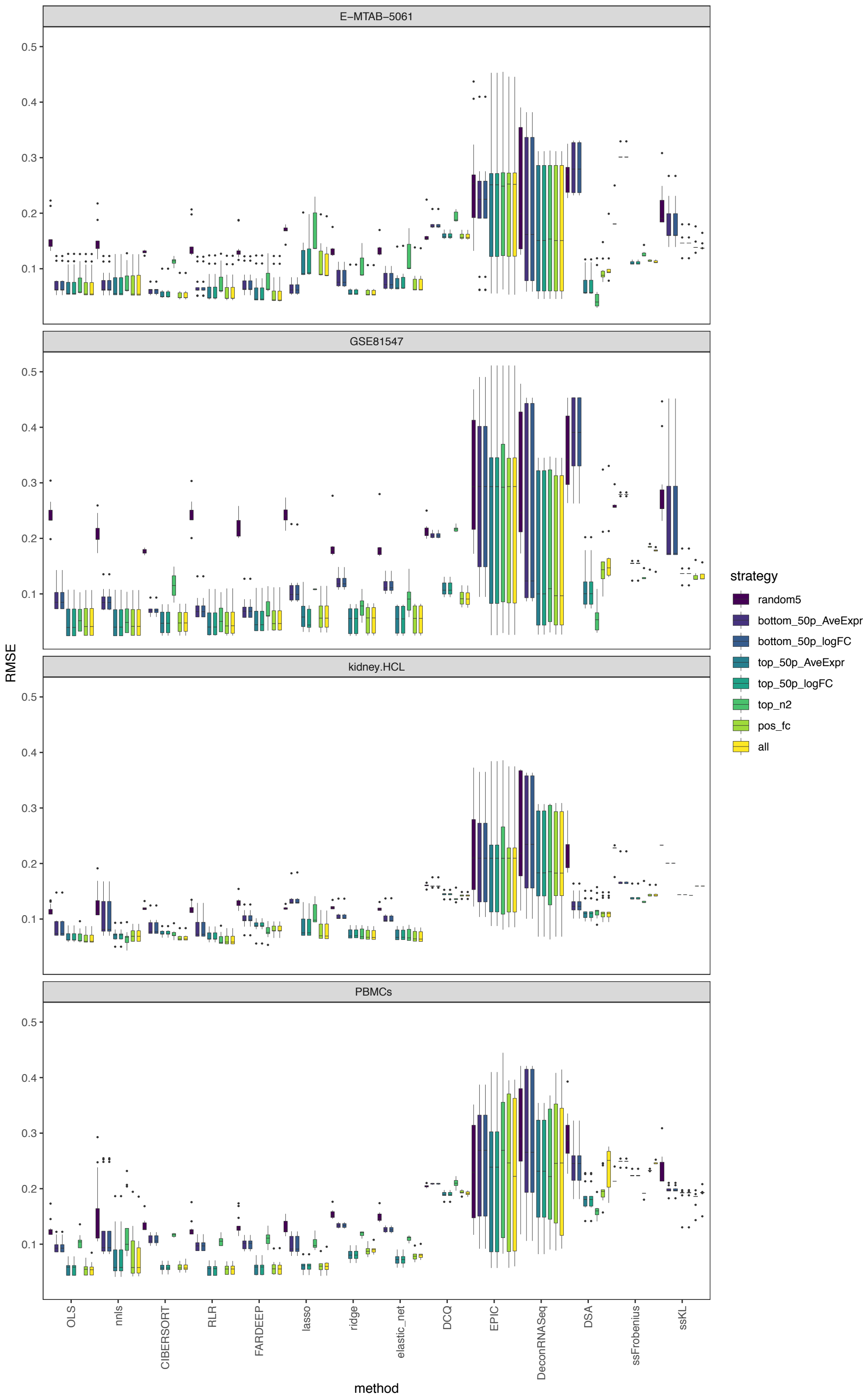

Supplementary Figure 13 – RMSE values between the expected (known) proportions in 1000 pseudo-bulk tissue mixtures (linear scale; pool size = 100 cells per mixture) and the output proportions from the E-MTAB-5061, GSE81547, kidney.HCL and PBMCs datasets, using eight different marker selection strategies. Each boxplot contains all normalization strategies that were tested in combination with a given marker strategy across the different bulk deconvolution methods.

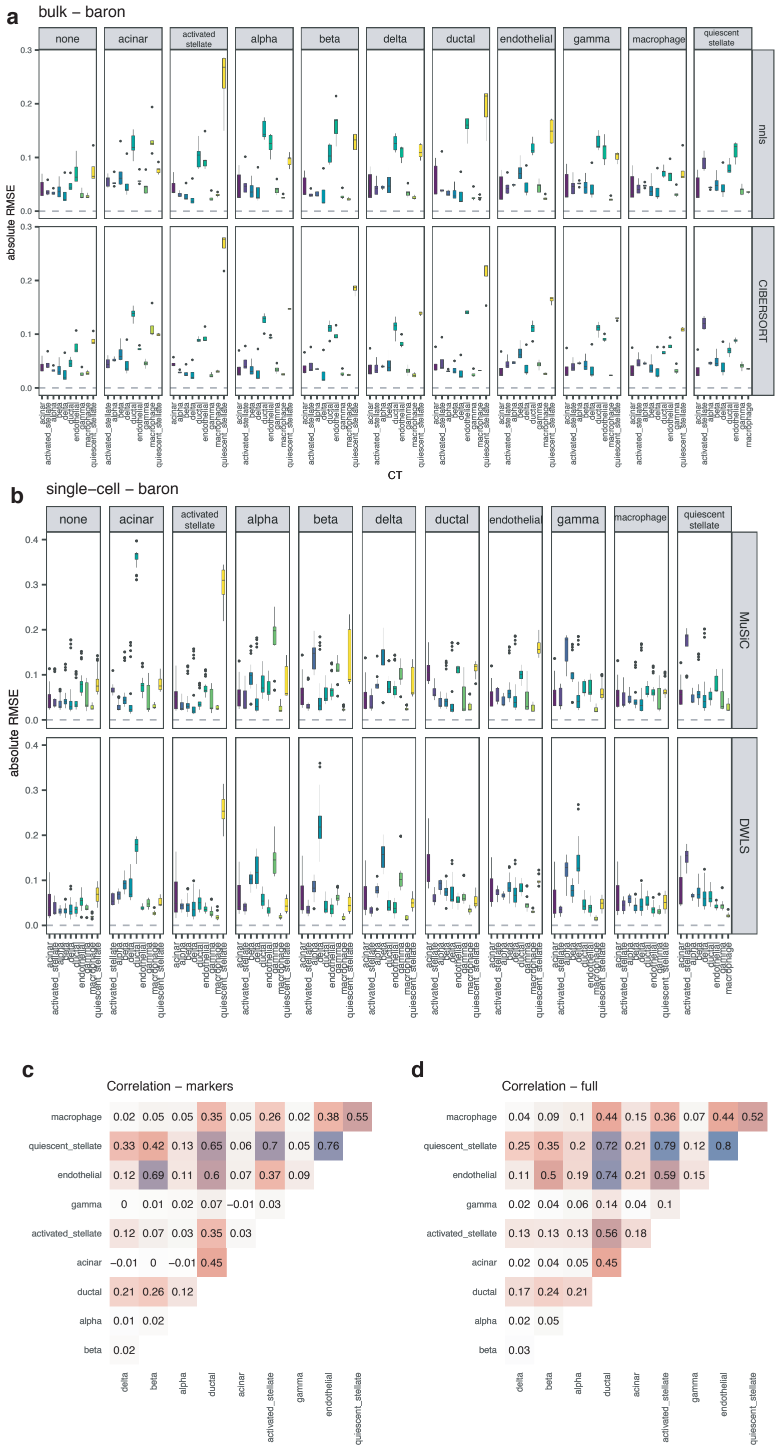

Supplementary Figure 14 – Effect of cell type removal on the deconvolution results using the baron dataset [100-cell pseudo-bulk mixtures in linear scale]. a) results using bulk deconvolution methods (nnls and CIBERSORT); b) results with deconvolution methods that use single-cell RNA-seq data as reference (MuSiC and DWLS); c) pairwise Pearson correlation values between expression profiles for the different cell types, using a subset of the reference matrix containing only the markers used in the bulk deconvolution; d) pairwise Pearson correlation values between complete expression profiles for the different cell types. In a) and b), each grey column represents a specific cell type removed. Each data point conforming a boxplot represents a different scaling/normalization strategy used.

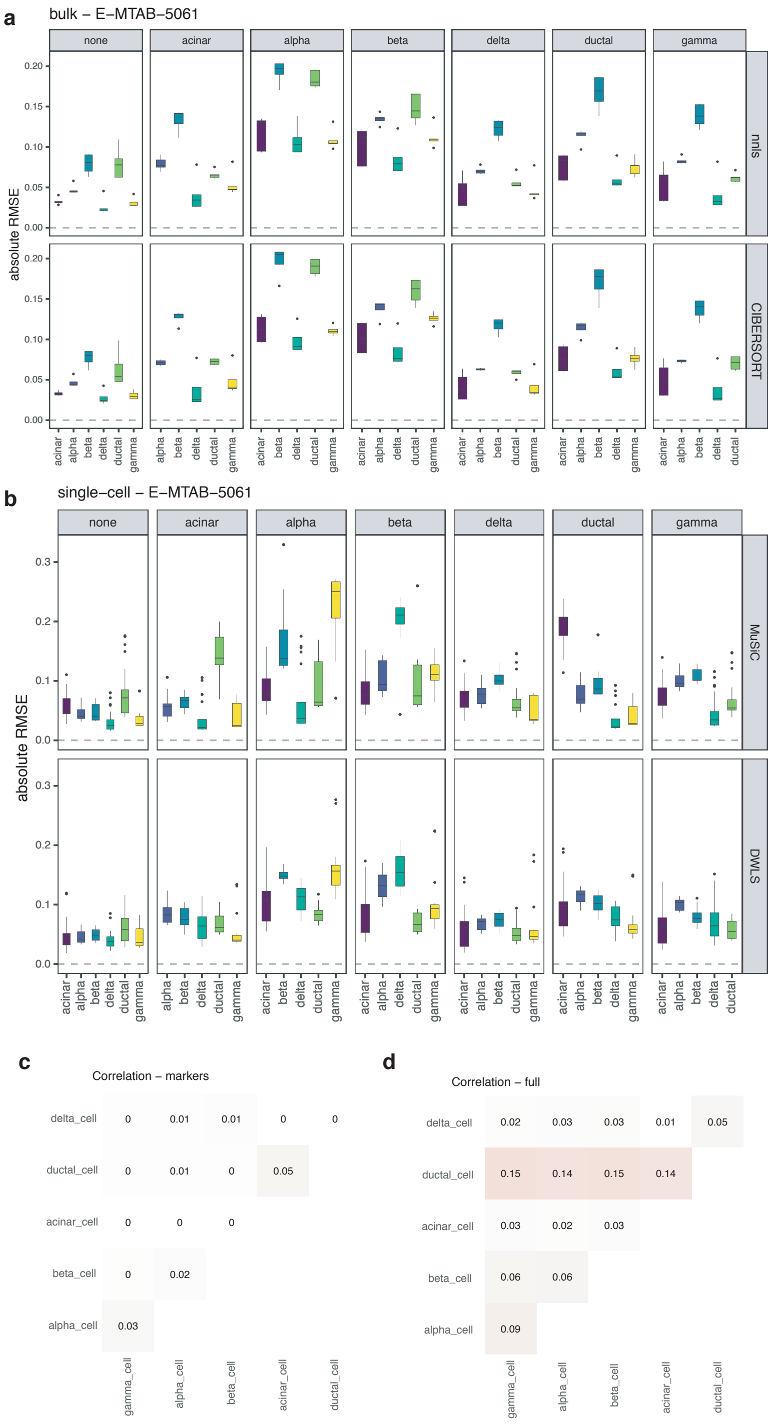

Supplementary Figure 15 – Effect of cell type removal on the deconvolution results using the E-MTAB-5061 dataset [100-cell pseudo-bulk mixtures in linear scale]. a) results using bulk deconvolution methods (nnls and CIBERSORT); b) results with deconvolution methods that use single-cell RNA-seq data as reference (MuSiC and DWLS); c) pairwise Pearson correlation values between expression profiles for the different cell types, using a subset of the reference matrix containing only the markers used in the bulk deconvolution; d) pairwise Pearson correlation values between complete expression profiles for the different cell types. In a) and b), each grey column represents a specific cell type removed. Each data point conforming a boxplot represents a different scaling/normalization strategy used.

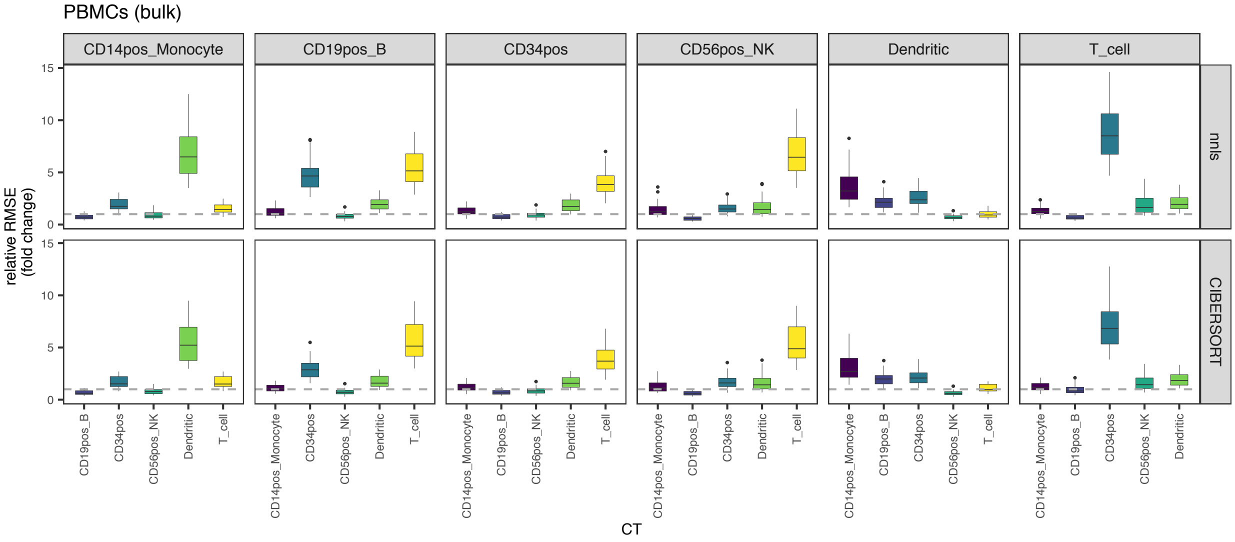

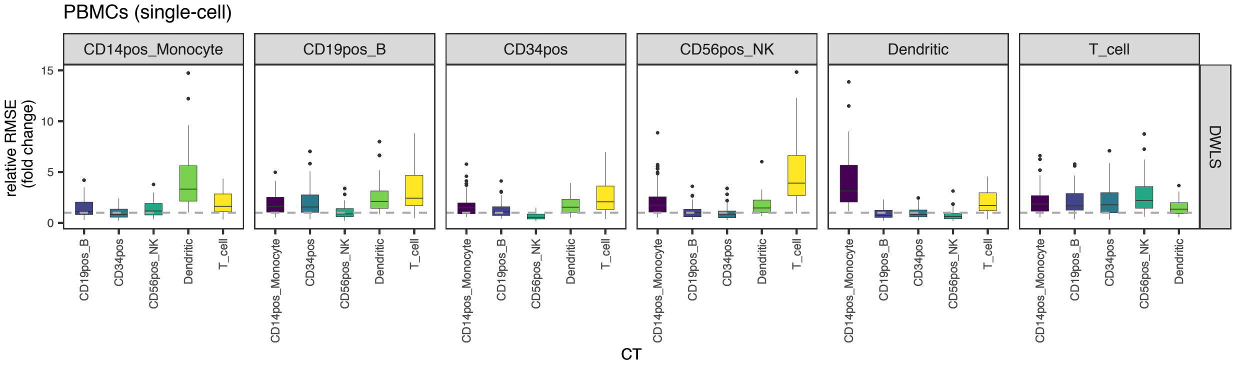

Supplementary Figure 16 – PBMCs dataset (linear scale; 1000 pseudo-bulk mixtures of 100 cells): RMSE fold change with respect to the ideal situation (=where all cell types in the mixtures were also present in the reference matrix) for both bulk deconvolution methods (top panel) and those that use scRNA-seq data as reference (bottom panel). The horizontal dashed line at fold change = 1 represents no changes observed, and values greater than one represent a detriment. Each grey column represents a specific cell type removed. Each data point conforming a boxplot represents a different scaling/normalization strategy used.

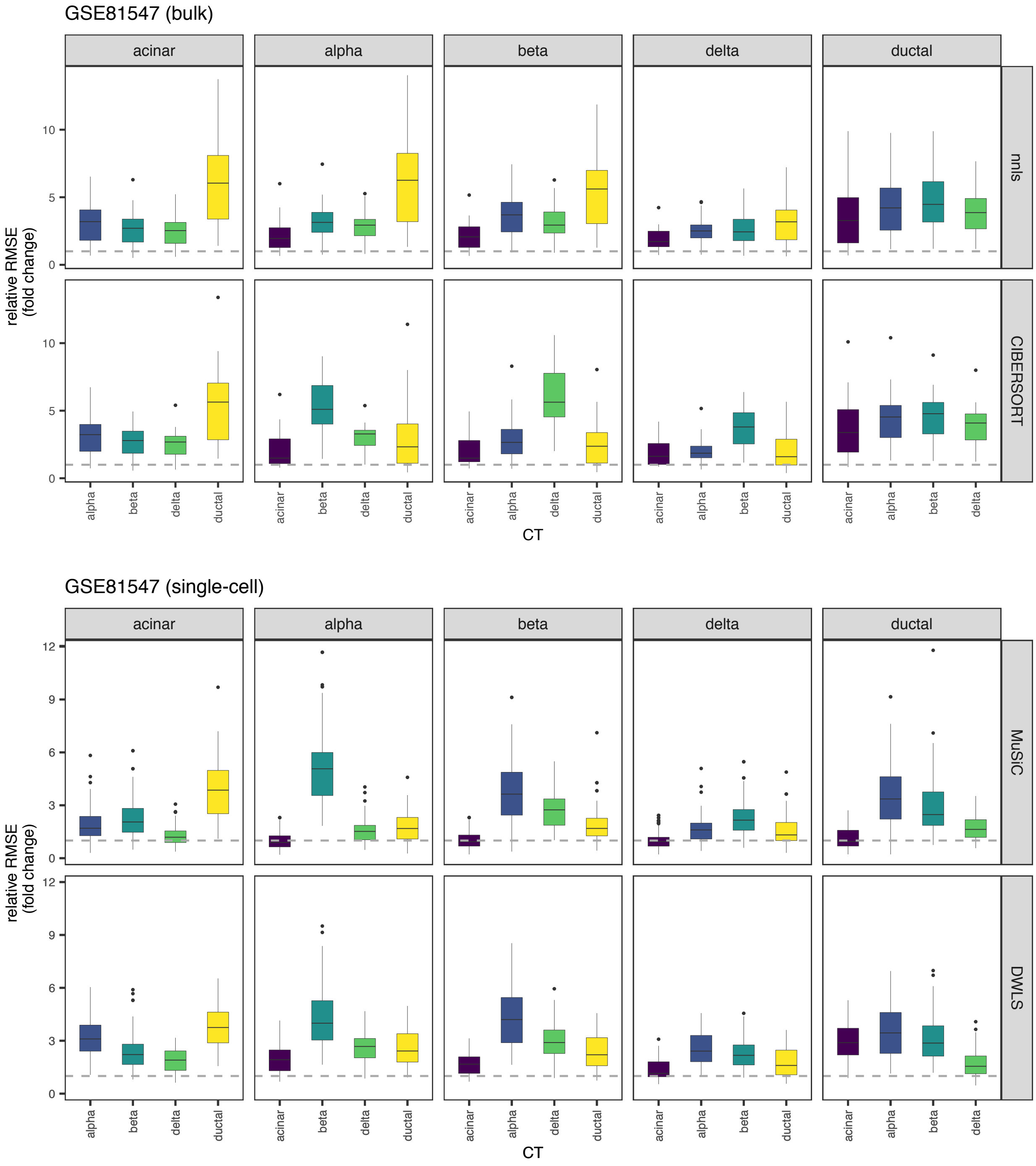

Supplementary Figure 17 – GSE81547 dataset (linear scale; 1000 pseudo-bulk mixtures of 100 cells): RMSE fold change with respect to the ideal situation (=where all cell types in the mixtures were also present in the reference matrix) for both bulk deconvolution methods (top panel) and those that use scRNA-seq data as reference (bottom panel). The horizontal dashed line at fold change = 1 represents no changes observed, and values greater than one represent a detriment. Each grey column represents a specific cell type removed. Each data point conforming a boxplot represents a different scaling/normalization strategy used.

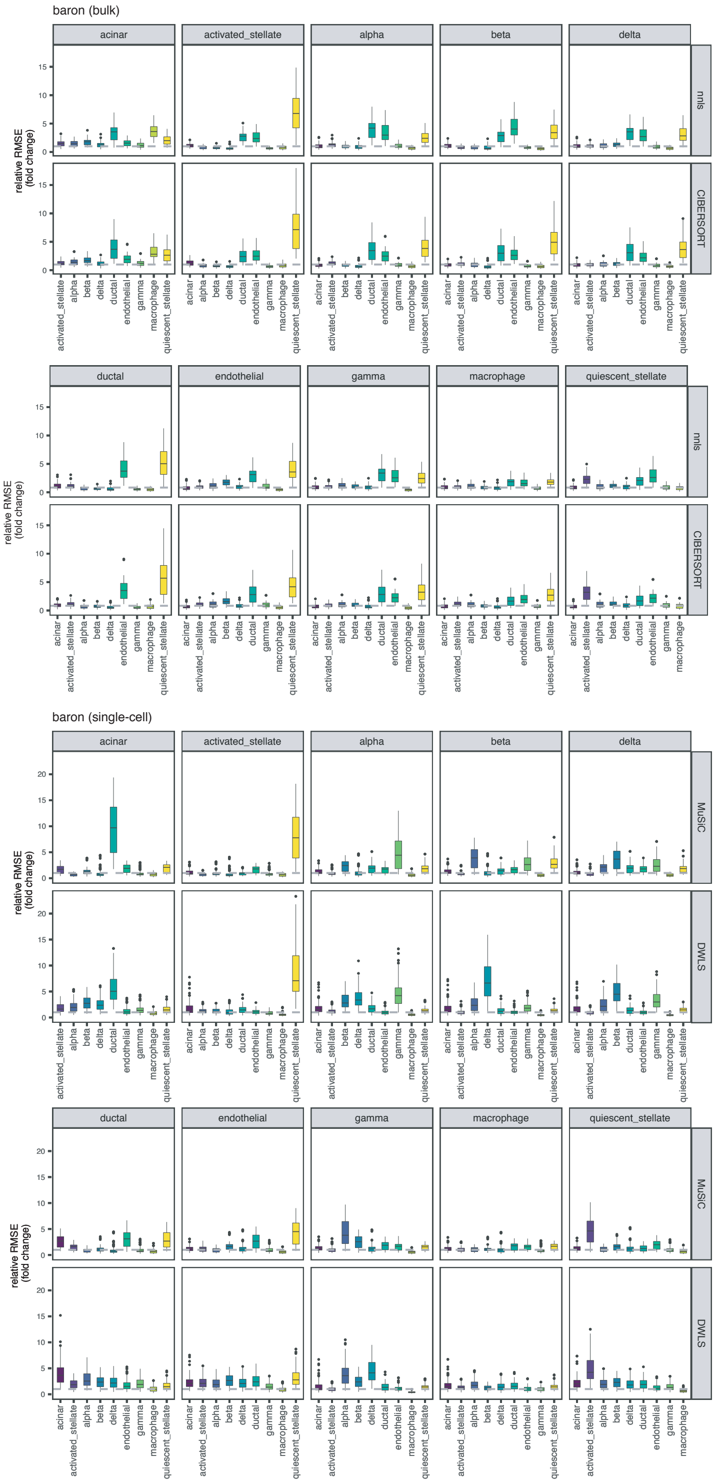

Supplementary Figure 18 – baron dataset (linear scale; 1000 pseudo-bulk mixtures of 100 cells): RMSE fold change with respect to the ideal situation (=where all cell types in the mixtures were also present in the reference matrix) for both bulk deconvolution methods (top panels) and those that use scRNA-seq data as reference (bottom panels). The horizontal dashed line at fold change = 1 represents no changes observed, and values greater than one represent a detriment. Each grey column represents a specific cell type removed. Each data point conforming a boxplot represents a different scaling/normalization strategy used.

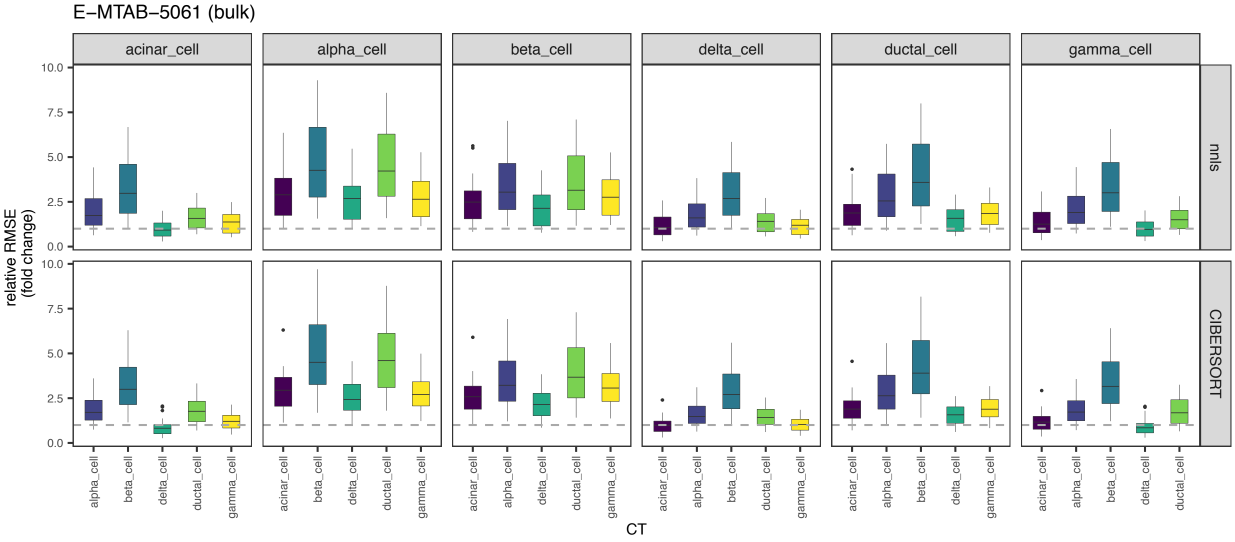

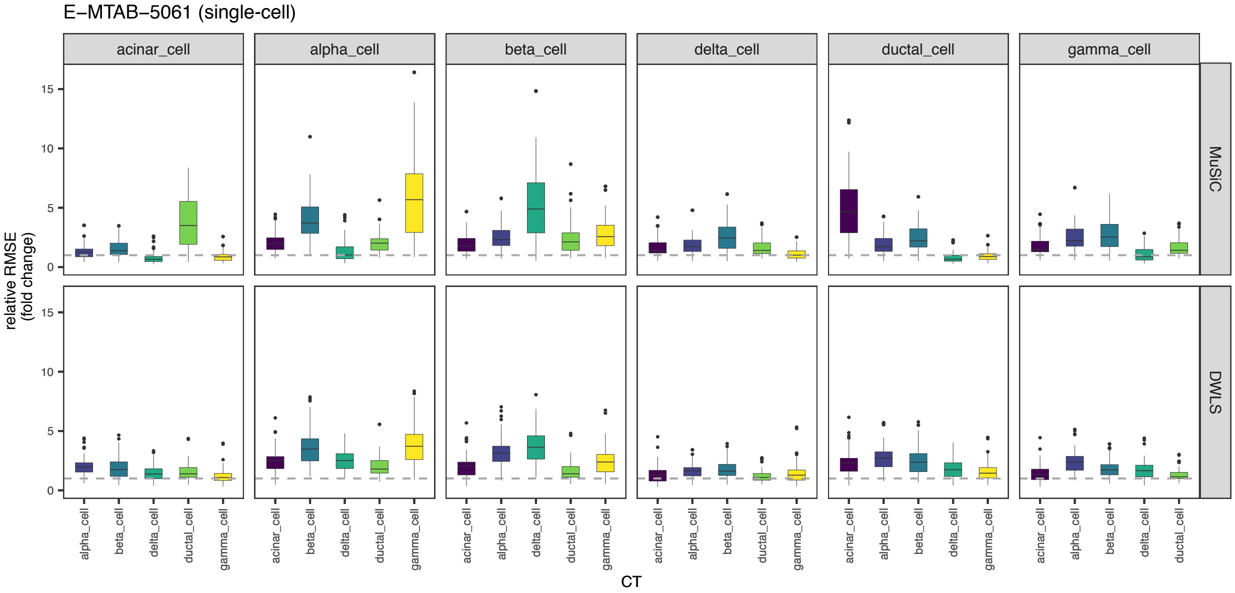

Supplementary Figure 19 – E-MTAB-5061 dataset (linear scale; 1000 pseudo-bulk mixtures of 100 cells): RMSE fold change with respect to the ideal situation (=where all cell types in the mixtures were also present in the reference matrix) for both bulk deconvolution methods (top panel) and those that use scRNA-seq data as reference (bottom panel). The horizontal dashed line at fold change = 1 represents no changes observed, and values greater than one represent a detriment. Each grey column represents a specific cell type removed. Each data point conforming a boxplot represents a different scaling/normalization strategy used.

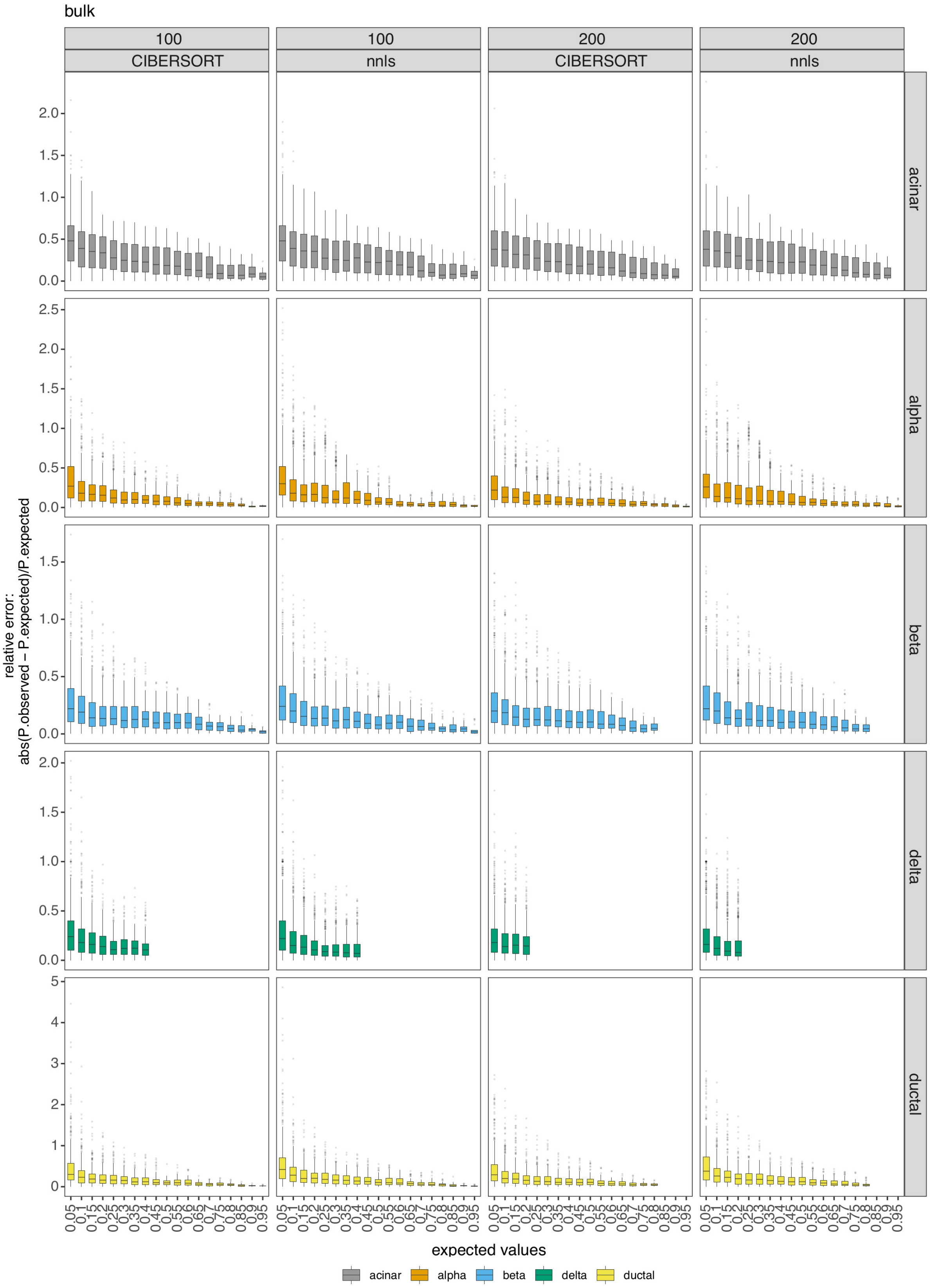

Supplementary Figure 20 – GSE81547 dataset: regardless of the cell type (each row), pseudo-bulk cell size (n = 100, 200) and bulk deconvolution method (nnls, CIBERSORT) being investigated, small proportions are always more difficult to reconstruct (higher relative errors) than high proportion values.

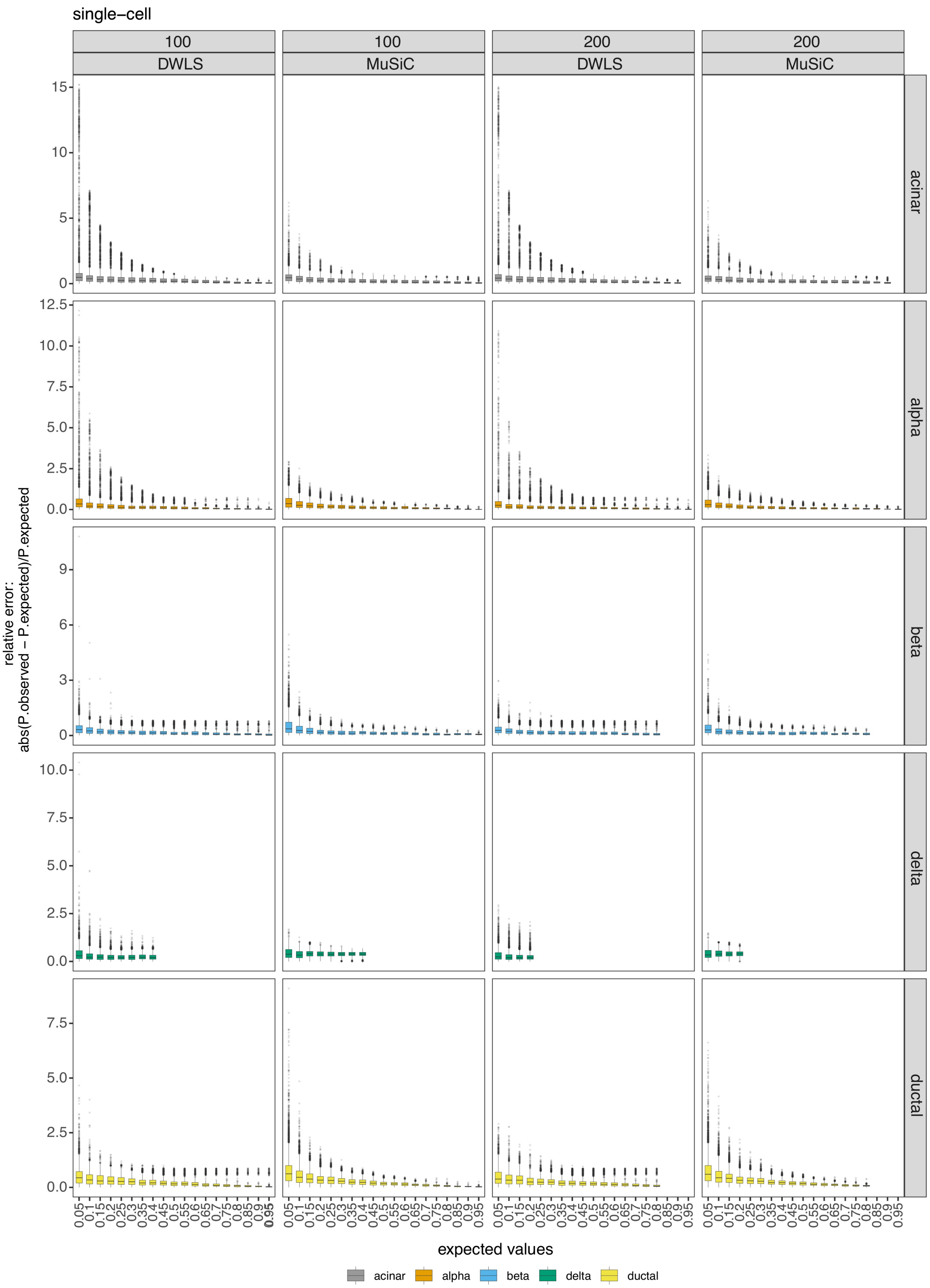

Supplementary Figure 21 – GSE81547 dataset: regardless of the cell type (each row), pseudo-bulk cell size (n = 100, 200) and deconvolution method that use scRNA-seq data as reference (DWLS, MuSiC) being investigated, small proportions are always more difficult to reconstruct (higher relative errors) than high proportion values.

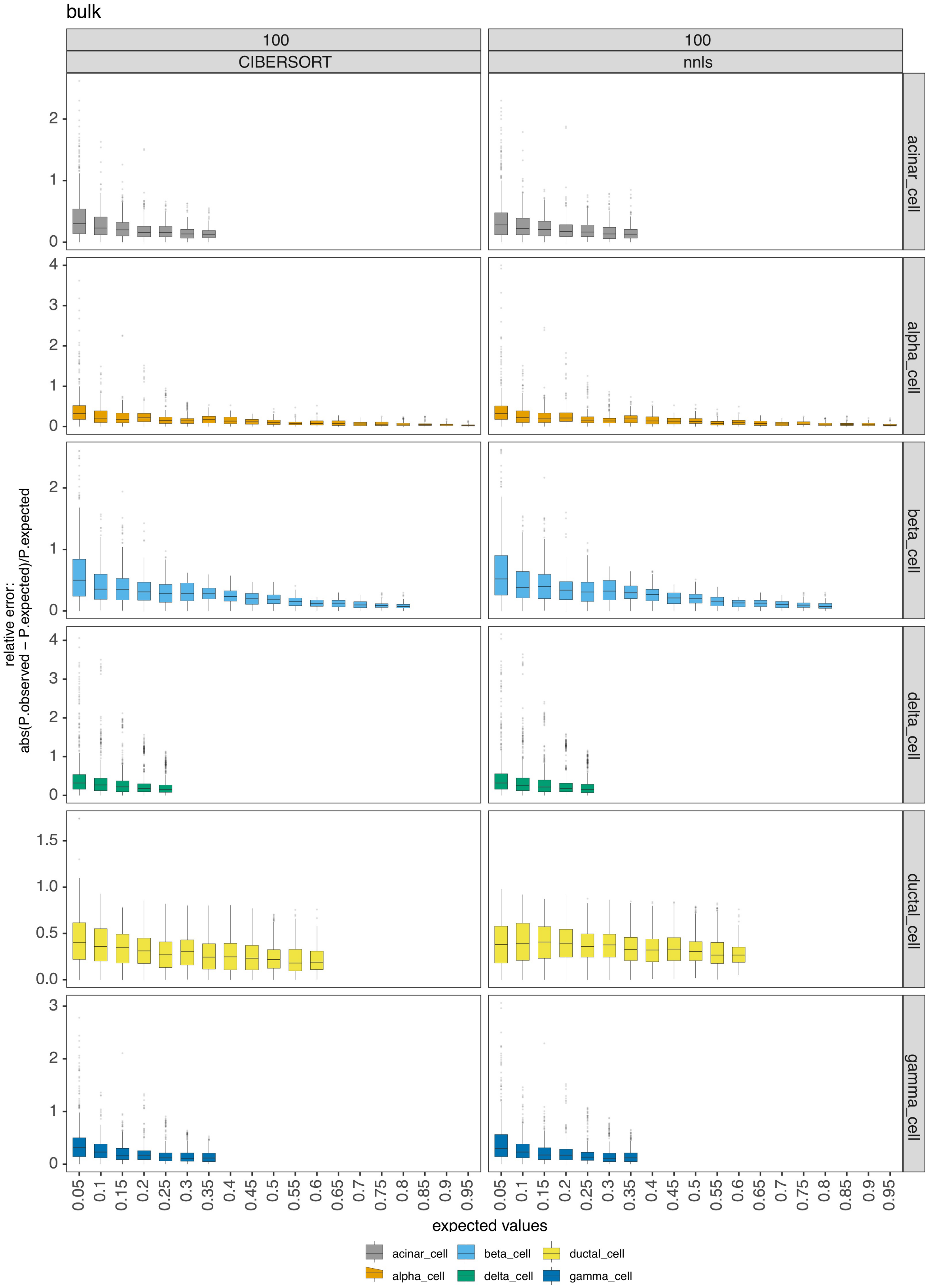

Supplementary Figure 22 – E-MTAB-5061 dataset: regardless of the cell type (each row), pseudo-bulk cell size (n = 100) and bulk deconvolution method (nnls, CIBERSORT) being investigated, small proportions are always more difficult to reconstruct (higher relative errors) than high proportion values.

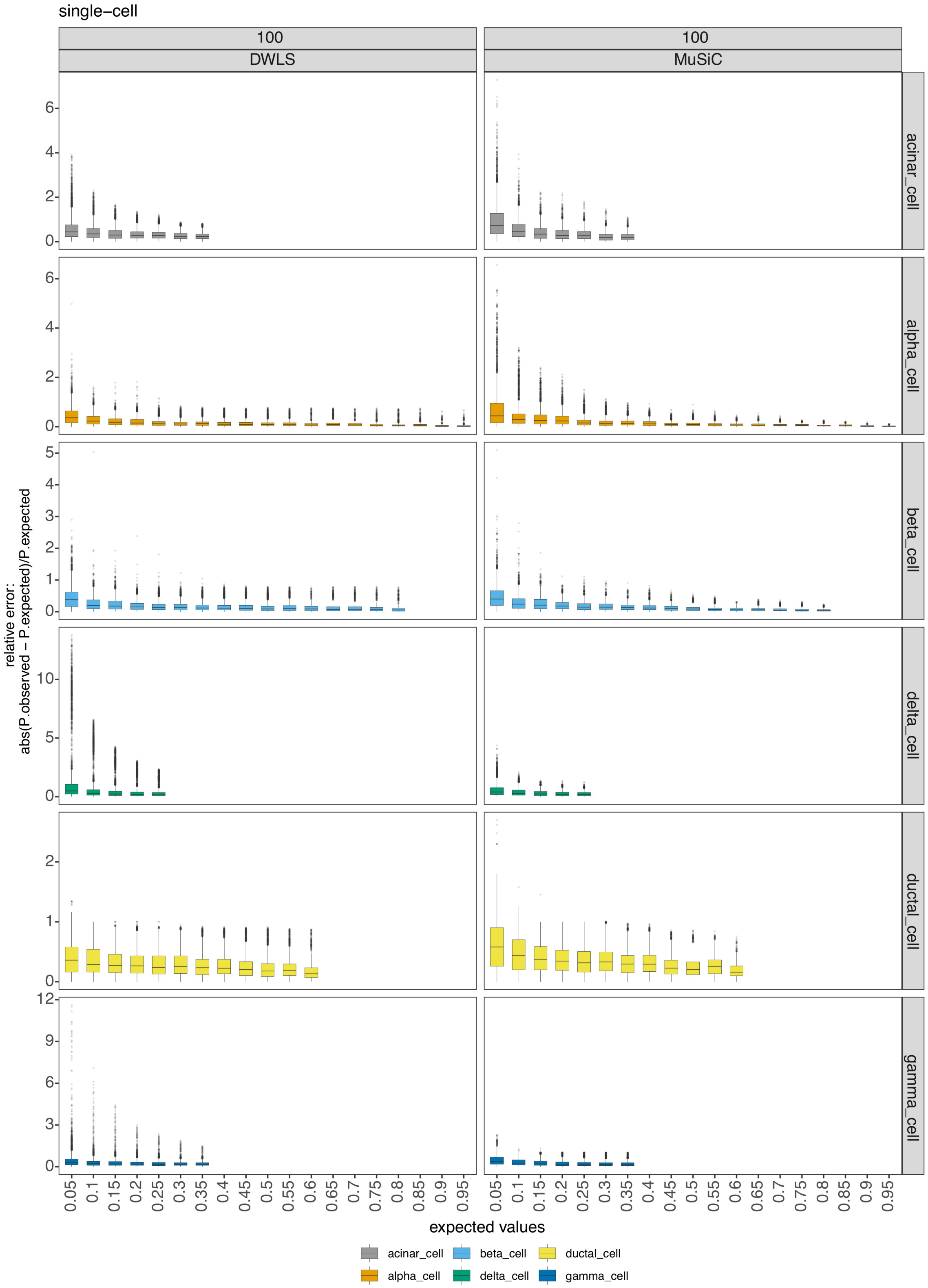

Supplementary Figure 23 – E-MTAB-5061 dataset: regardless of the cell type (each row), pseudo-bulk cell size (n = 100) and deconvolution method that use scRNA-seq data as reference (DWLS, MuSiC) being investigated, small proportions are always more difficult to reconstruct (higher relative errors) than high proportion values.

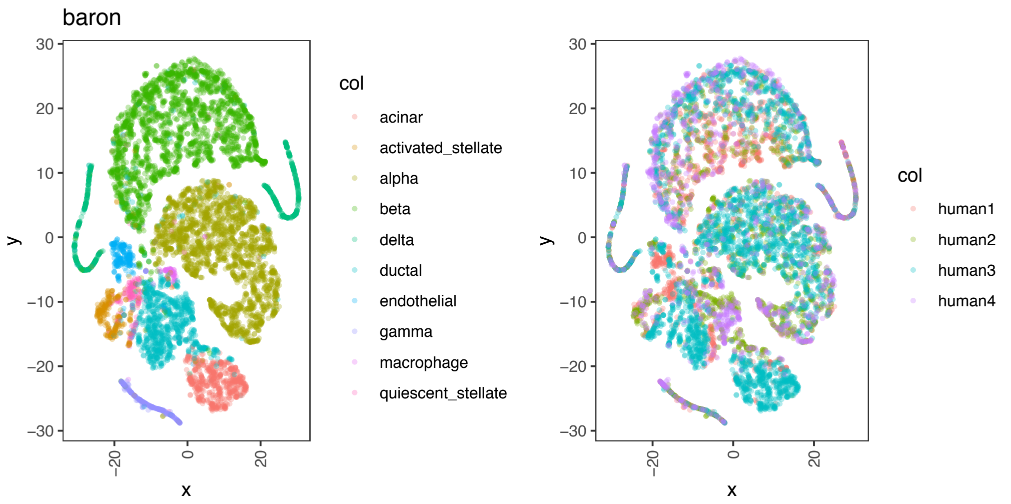
baron
col
acinar
activated_stellate
alpha
beta
delta
ductal
endothelial
gamma
macrophage
quiescent_stellate
col
human1
human2
human3
human4
x
y

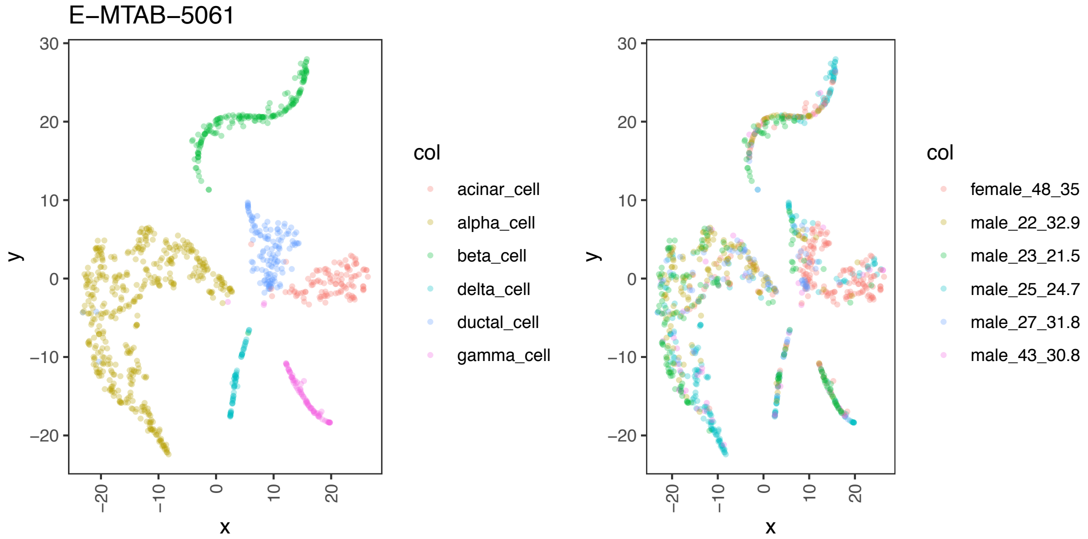
E−MTAB−5061
col
acinar_cell
alpha_cell
beta_cell
delta_cell
ductal_cell
gamma_cell
col
female_48_35
male_22_32.9
male_23_21.5
male_25_24.7
male_27_31.8
male_43_30.8
x
y

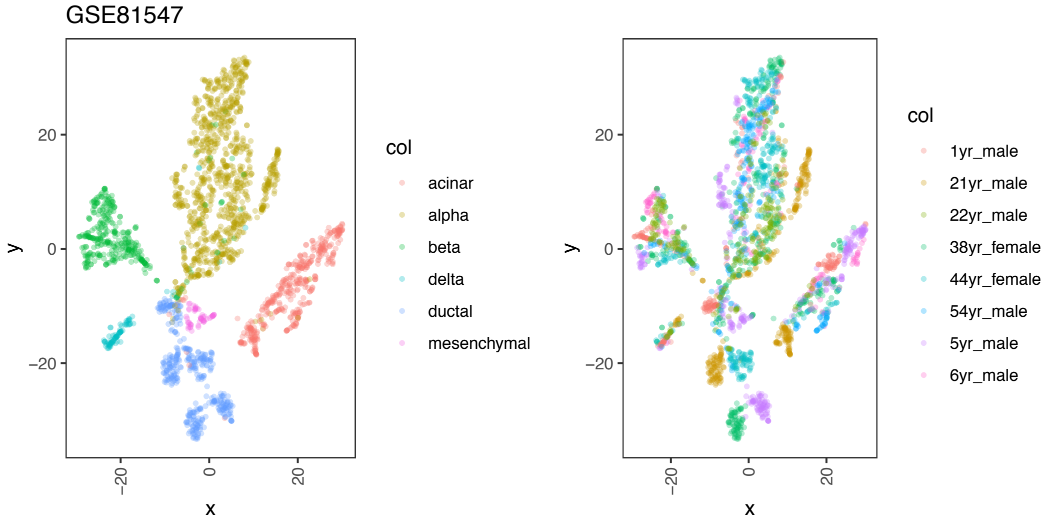
GSE81547
col
acinar
alpha
beta
delta
ductal
mesenchymal
col
1yr_male
21yr_male
22yr_male
38yr_female
44yr_female
54yr_male
5yr_male
6yr_male
x
y

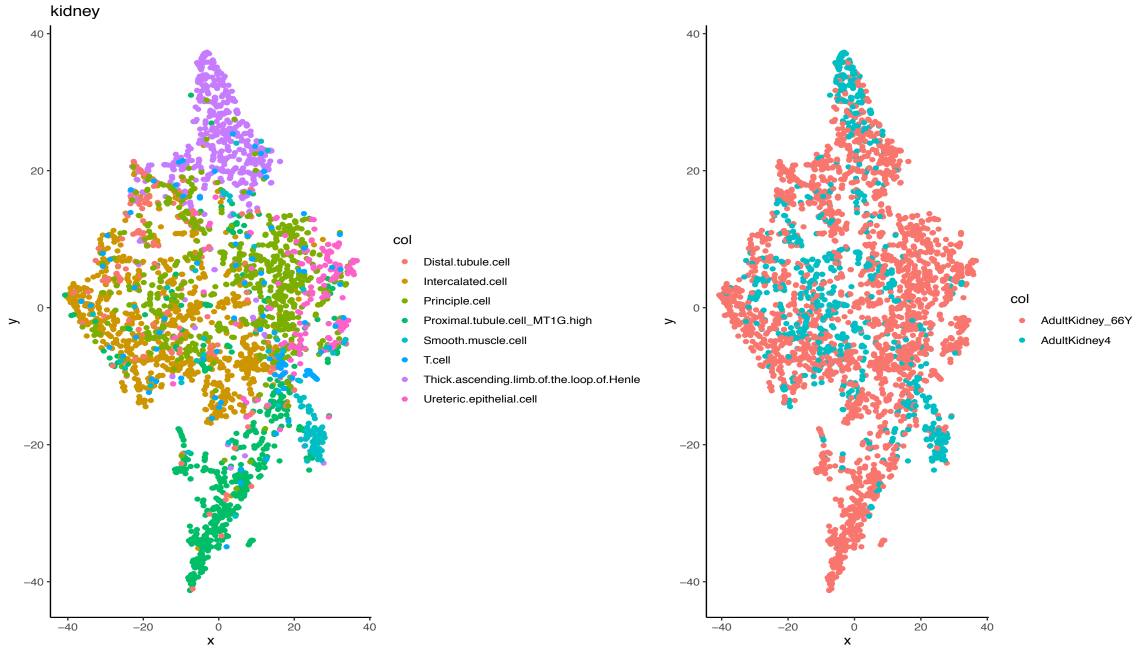

Supplementary Figure 24 – Dimensionality reduction plots (tSNE) by cell type (left) and donor (right) across datasets after quality control.

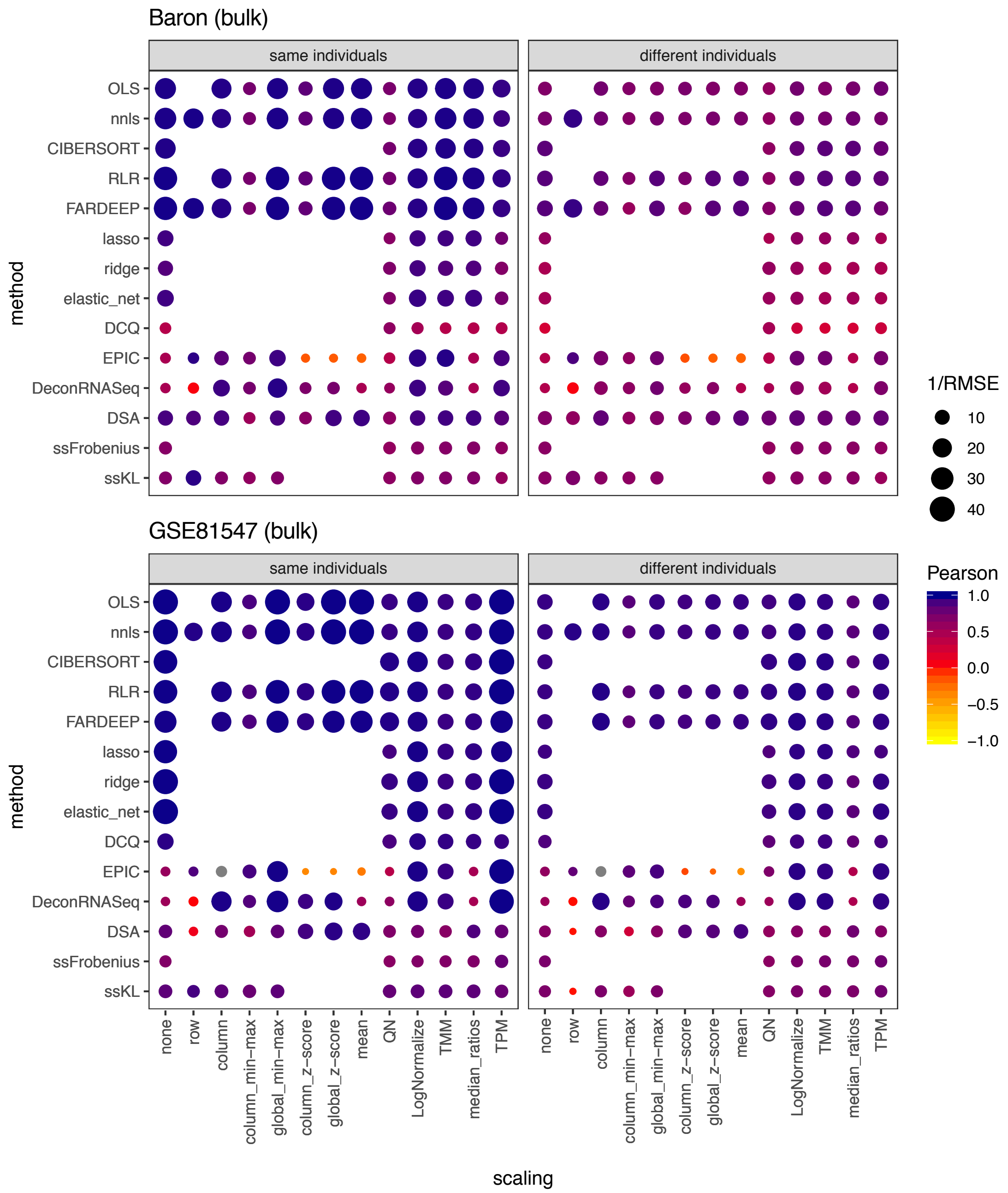

Supplementary Figure 25 – RMSE and Pearson correlation values between the expected (known) proportions in 1000 pseudo-bulk tissue mixtures in linear scale (pool size = 100 cells per mixture) and the output proportions from the different bulk deconvolution methods for Baron and GSE81547 datasets (top and bottom panel, respectively). “Same individuals” represent scenarios where both training and test sets contained cells from all individuals whereas “different individuals” represent results where training and test splits were made of cells from independent individuals.

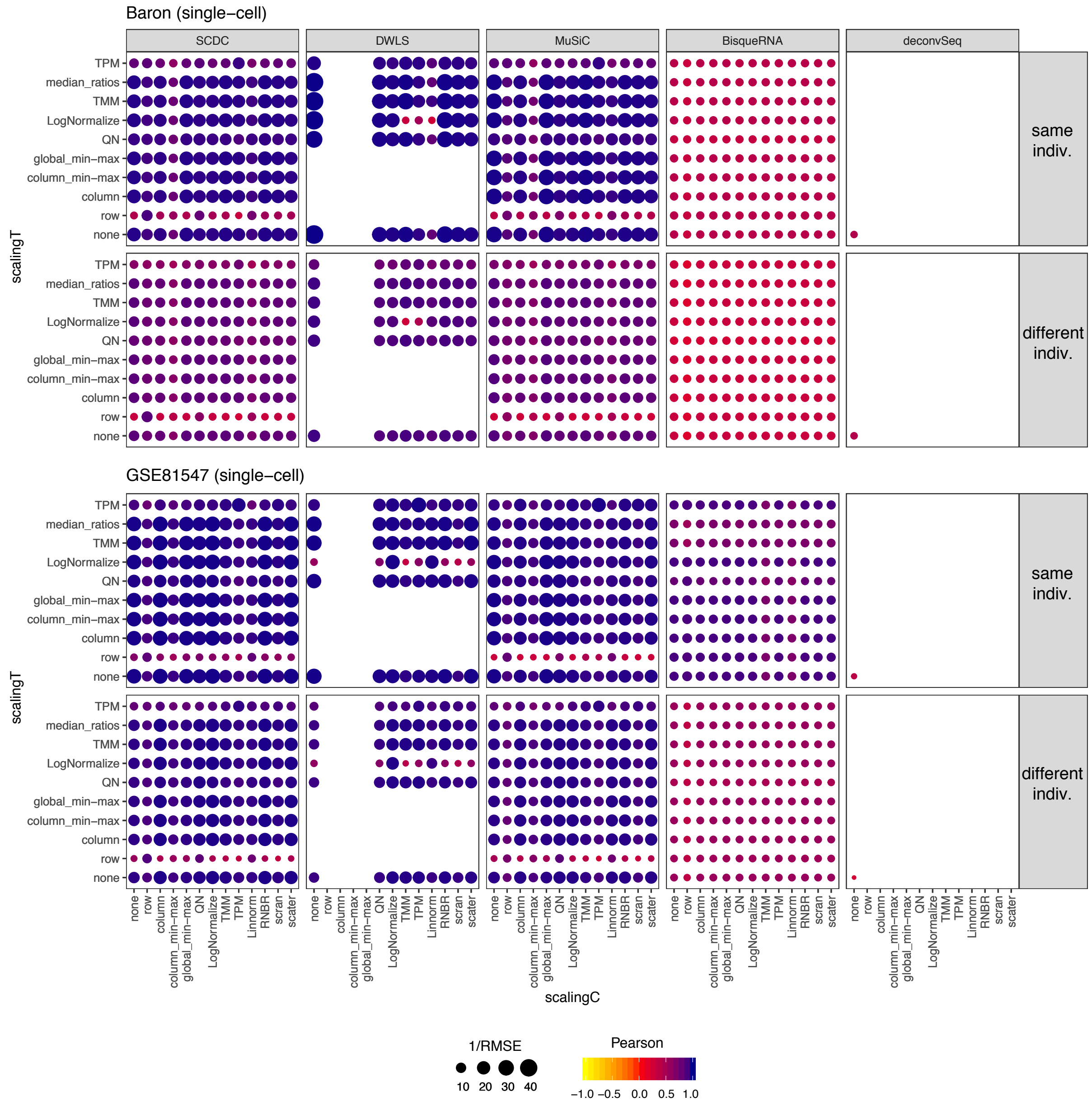

Supplementary Figure 26 – RMSE and Pearson correlation values between the expected (known) proportions in 1000 pseudo-bulk tissue mixtures in linear scale (pool size = 100 cells per mixture) and the output proportions from the different deconvolution methods that use scRNA-seq data as input (for Baron and GSE81547 datasets; top and bottom panel, respectively). “Same individ.” represent scenarios where both training and test sets contained cells from all individuals whereas “different indiv.” represent results where training and test splits were made of cells from independent individuals.

SUPPLEMENTARY REFERENCES

1. Scialdone, A. *et al.* Computational assignment of cell-cycle stage from single-cell transcriptome data. *Methods* 85, 54–61 (2015).
2. Finak, G. *et al.* MAST: a flexible statistical framework for assessing transcriptional changes and characterizing heterogeneity in single-cell RNA sequencing data. *Genome Biol.* 16, 278 (2015).
