# Supplementary material for: Benchmarking of cell type deconvolution pipelines for transcriptomics data
Source: Nat Commun. 2020 Nov 6;11:5650. doi: 10.1038/s41467-020-19015-1 (PMC7648640; doi:10.1038/s41467-020-19015-1)
Supplement: Supplementary file 3 — Reporting Summary [file 41467_2020_19015_MOESM3_ESM.pdf]

nature research

Corresponding author(s): Katleen De Preter

Last updated by author(s): Aug 13, 2020

Reporting Summary

Nature Research wishes to improve the reproducibility of the work that we publish. This form provides structure for consistency and transparency in reporting. For further information on Nature Research policies, see our Editorial Policies and the Editorial Policy Checklist.

Statistics

For all statistical analyses, confirm that the following items are present in the figure legend, table legend, main text, or Methods section.

n/a	Confirmed	
☐	☒	The exact sample size (*n*) for each experimental group/condition, given as a discrete number and unit of measurement
☐	☒	A statement on whether measurements were taken from distinct samples or whether the same sample was measured repeatedly
☒	☐	The statistical test(s) used AND whether they are one- or two-sided<br>*Only common tests should be described solely by name; describe more complex techniques in the Methods section.*
☐	☒	A description of all covariates tested
☒	☐	A description of any assumptions or corrections, such as tests of normality and adjustment for multiple comparisons
☒	☐	A full description of the statistical parameters including central tendency (e.g. means) or other basic estimates (e.g. regression coefficient) AND variation (e.g. standard deviation) or associated estimates of uncertainty (e.g. confidence intervals)
☒	☐	For null hypothesis testing, the test statistic (e.g. *F*, *t*, *r*) with confidence intervals, effect sizes, degrees of freedom and *P* value noted<br>*Give P values as exact values whenever suitable.*
☒	☐	For Bayesian analysis, information on the choice of priors and Markov chain Monte Carlo settings
☒	☐	For hierarchical and complex designs, identification of the appropriate level for tests and full reporting of outcomes
☒	☐	Estimates of effect sizes (e.g. Cohen's *d*, Pearson's *r*), indicating how they were calculated

Our web collection on statistics for biologists contains articles on many of the points above.

Software and code

Policy information about availability of computer code

Data collection	Publicly available datasets where directly downloaded from their sources
Data analysis	Source code can be found at https://github.com/favilaco/deconv_benchmark; Software used: STAR v2.6.0c; R statistical programming language (v3.6.2) along with the packages: BisqueRNA_1.0.1; deconvSeq_0.1.2; SCDC_0.0.0.9000; DWLS_0.1; varhandle_2.0.4; ROCR_1.0-7; gplots_3.0.1.1; e1071_1.7-3; quadprog_1.5-8; reshape2_1.4.3; MuSiC_0.1.1; EPIC_1.1.5; DSA_1.0; CellMix_1.6.2; MAST_1.10.0; genefilter_1.68.0; GSEABase_1.48.0; graph_1.64.0; annotate_1.64.0; XML_3.98-1.20; AnnotationDbi_1.46.1; multtest_2.40.0; DeconRNASeq_1.28.0; Linnorm_2.10.0; scater_1.12.2; SingleCellExperiment_1.6.0; SingleR_1.0.6; preprocessCore_1.48.0; pcaMethods_1.78.0; DESeq2_1.26.0; SummarizedExperiment_1.14.1; DelayedArray_0.10.0; BiocParallel_1.20.1; GenomicRanges_1.36.1; GenomeInfoDb_1.20.0; IRanges_2.20.1; S4Vectors_0.24.1; edgeR_3.26.8; limma_3.40.6; corpcor_1.6.9; limSolve_1.5.6; csSAM_1.2.4; NMF_0.21.0; Biobase_2.46.0; BiocGenerics_0.32.0; cluster_2.1.0; rngtools_1.4; pkgmaker_0.28; registry_0.5-1; dtangle_2.0.9; ComICS_1.0.4; glmnet_3.0-2; MASS_7.3-51.5; FARDEEP_1.0.1; nnls_1.4; sctransform_0.2.1; Seurat_3.1.2; doSNOW_1.0.18; snow_0.4-3; doMC_1.3.6; iterators_1.0.12; foreach_1.4.7; gtools_3.8.1; matrixStats_0.55.0; Matrix_1.2-18; forcats_0.4.0; stringr_1.4.0; dplyr_0.8.3; purrr_0.3.3; readr_1.3.1; tidyr_1.0.0; tibble_3.0.3; tidyverse_1.3.0; ggplot2_3.2.1; data.table_1.12.8; BiocManager_1.30.10; devtools_2.2.1; usethis_1.5.1; pbapply_1.4-2; lattice_0.20-38; haven_2.3.1; vctrs_0.3.2; methylKit_1.10.0; mgcv_1.8-31; blob_1.2.0; survival_3.1-8; later_1.0.0; nloptr_1.2.1; DBI_1.1.0; R.utils_2.9.2; rappdirs_0.3.1; uwot_0.1.5; dqrng_0.2.1; jpeg_0.1-8.1; zlibbioc_1.32.0; MatrixModels_0.4-1; SDMTools_1.1-221.2; htmlwidgets_1.5.1; mvtnorm_1.0-11; future_1.15.1; leiden_0.3.1; irlba_2.3.3; DEoptimR_1.0-8; Rcpp_1.0.3; KernSmooth_2.23-16; promises_1.1.0; gdata_2.18.0; vegan_2.5-6; pkgload_1.0.2; RcppParallel_4.4.4; Hmisc_4.3-0; apcluster_1.4.8; fs_1.3.1; mnormt_1.5-5; digest_0.6.23; png_0.1-7; cowplot_1.0.0; pkgconfig_2.0.3; gridBase_0.4-7; DelayedMatrixStats_1.6.1; ggbeeswarm_0.6.0; minqa_1.2.4; emdbook_1.3.11; reticulate_1.14; beeswarm_0.2.3; modeltools_0.2-23; xfun_0.11; zoo_1.8-6; tidyselect_0.2.5; kernlab_0.9-29; ica_1.0-2; viridisLite_0.3.0; rtracklayer_1.44.4; pkgbuild_1.0.6; rlang_0.4.7; simpleSingleCell_1.8.0; glue_1.3.1; fastseg_1.30.0; metap_1.2; RColorBrewer_1.1-2; modelr_0.1.8; fpc_2.2-7; SparseM_1.78; gbRd_0.4-11; mutoss_0.1-12; httpuv_1.5.2; class_7.3-15; BiocNeighbors_1.2.0; TH.data_1.0-10; jsonlite_1.6; XVector_0.24.0; bit_1.1-14; mime_0.8; gridExtra_2.3; Rsamtools_2.0.3; BiocStyle_2.12.0; stringi_1.4.3; gmodels_2.18.1; processx_3.4.1; bitops_1.0-6; cli_2.0.0; Rdpack_0.11-1; RSQLite_2.1.5; pheatmap_1.0.12; rstudioapi_0.10;

GenomicAlignments_1.20.1; nlme_3.1-143; qvalue_2.16.0; scran_1.12.1; fastcluster_1.1.25; locfit_1.5-9.1; listenv_0.8.0; lpSolve_5.6.13.3; R.oo_1.23.0; prabclus_2.3-2; dbplyr_1.4.2; sessioninfo_1.1.1; readxl_1.3.1; lifecycle_0.2.0; ExperimentHub_1.12.0; munsell_0.5.0; cellranger_1.1.0; R.methodsS3_1.7.1; caTools_1.17.1.3; codetools_0.2-16; coda_0.19-3; vipor_0.4.5; lmtest_0.9-37; htmlTable_1.13.3; lsei_1.2-0; xtable_1.8-4; diptest_0.75-7; abind_1.4-5; AnnotationHub_2.18.0; RANN_2.6.1; bibtex_0.4.2.1; RcppAnnoy_0.0.14; ggdendro_0.1.21; future.apply_1.3.0; ellipsis_0.3.0; prettyunits_1.0.2; L1pack_0.38.19; lubridate_1.7.9; ggridges_0.5.1; reprex_0.3.0; mclust_5.4.5; igraph_1.2.4.2; remotes_2.1.0; TFisher_0.2.0; testthat_2.3.1; htmltools_0.4.0; BiocFileCache_1.10.2; Rmisc_1.5; yaml_2.2.0; MCMCpack_1.4-5; plotly_4.9.1; interactiveDisplayBase_1.24.0; foreign_0.8-74; withr_2.1.2; fitdistrplus_1.0-14; bit64_0.9-7; multcomp_1.4-11; robustbase_0.93-6; Biostrings_2.52.0; rsvd_1.0.2; evaluate_0.14; memoise_1.1.0; geneplotter_1.64.0; permute_0.9-5; callr_3.4.0; ps_1.3.0; curl_4.3; fansi_0.4.0; acepack_1.4.1; checkmate_1.9.4; desc_1.2.0; npsurv_0.4-0; truncnorm_1.0-8; ellipse_0.4.2; ggrepel_0.8.1; rprojroot_1.3-2; tools_3.6.2; sandwich_2.5-1; magrittr_1.5; Rsolnp_1.16; RCurl_1.95-4.12; ape_5.3; xml2_1.2.2; rmarkdown_2.0; httr_1.4.1; assertthat_0.2.1; boot_1.3-24; globals_0.12.5; R6_2.4.1; nnet_7.3-12; progress_1.2.2; shape_1.4.4; statmod_1.4.32; BiocVersion_3.10.1; BiocSingular_1.0.0; splines_3.6.2; colorspace_1.4-1; amap_0.8-18; generics_0.0.2; base64enc_0.1-3; pillar_1.4.3; sn_1.5-4; GenomeInfoDbData_1.2.2; plyr_1.8.5; gtable_0.3.0; bdsmatrix_1.3-3; rvest_0.3.6; psych_1.9.12; knitr_1.26; RcppArmadillo_0.9.800.3.0; latticeExtra_0.6-29; biomaRt_2.40.5; fastmap_1.0.1; BiocInstaller_1.30.0; doParallel_1.0.15; flexmix_2.3-15; quantreg_5.54; broom_0.7.0; scales_1.1.0; backports_1.1.5; plotrix_3.7-7; lme4_1.1-21; blme_1.0-4; mcmc_0.9-6; hms_0.5.2; Rtsne_0.15; shiny_1.4.0; numDeriv_2016.8-1.1; bbmle_1.0.22; lazyeval_0.2.2; dynamicTreeCut_1.63-1; Formula_1.2-3; tsne_0.1-3; crayon_1.3.4; viridis_0.5.1; rpart_4.1-15

For manuscripts utilizing custom algorithms or software that are central to the research but not yet described in published literature, software must be made available to editors and reviewers. We strongly encourage code deposition in a community repository (e.g. GitHub). See the Nature Research guidelines for submitting code & software for further information.

Data

Policy information about availability of data

All manuscripts must include a data availability statement. This statement should provide the following information, where applicable:

- Accession codes, unique identifiers, or web links for publicly available datasets
- A list of figures that have associated raw data
- A description of any restrictions on data availability

The five publicly available datasets used in this article can be found at their respective sources:
Baron: https://www.ncbi.nlm.nih.gov/geo/query/acc.cgi?acc=GSE84133 (Specifically, GSM2230757 to GSM2230760 for human pancreatic islands (healthy, non-diabetic))
GSE81547: https://www.ncbi.nlm.nih.gov/geo/query/acc.cgi?acc=GSE81547 (healthy, non-diabetic)
E-MTAB-5061: https://www.ebi.ac.uk/arrayexpress/experiments/E-MTAB-5061/ (only healthy, non-diabetic patients were used)
PBMCs: https://support.10xgenomics.com/single-cell-gene-expression/datasets/1.1.0/fresh_68k_pbmc_donor_a (healthy donor A)
kidney.HCL: https://figshare.com/articles/HCL_DGE_Data/7235471 (Adult Kidney2 and Adult Kidney4)

Field-specific reporting

Please select the one below that is the best fit for your research. If you are not sure, read the appropriate sections before making your selection.

☒ Life sciences ☐ Behavioural & social sciences ☐ Ecological, evolutionary & environmental sciences

For a reference copy of the document with all sections, see nature.com/documents/nr-reporting-summary-flat.pdf

Life sciences study design

All studies must disclose on these points even when the disclosure is negative.

Sample size	We used five publicly available scRNA-seq datasets containing up to 10,000 individual cells and coming from 1 to 8 different individuals (see Table 1). Since testing all possible combinations of all factors evaluated in this study was computationally intensive (=millions of combinations) and thus a bottleneck, the number of datasets was restricted to five. Other than that, no sample-size calculation was used.
Data exclusions	Only cells with known cell type labels were used. For this reason, regarding E-MTAB-5061, cells with "not_applicable", "unclassified" and "co-expression_cell" labels were excluded (otherwise it is not possible to know the composition of the mixtures). Furthermore, since the other two pancreatic datasets (Baron and GSE81547) only contain healthy non-diabetic samples, only cells coming from six healthy patients (non-diabetic) were kept in E-MTAB-5061.
Replication	All bulk deconvolution methods and three out of five method using single-cell RNA-seq data as input (DWLS, BisqueRNA and deconvSeq) were tested and compared in all 5 different (independent) datasets, obtaining similar results. MuSiC and SCDC required scRNA-seq from at least two different samples to perform the deconvolution, so they were not tested in the PBMC dataset (n=1).
Randomization	We retained cell types with at least 50 cells passing the quality control step and, by setting a fixed seed and taking into account the number of cells across the different cell types (pooling different individuals when possible; thereby including inherent inter-sample variability), each dataset was further split into balanced "training" and "testing" datasets (50%:50% split) with a similar distribution of cells per cell type. Given the limited number of cells available per dataset and the scarcity of publicly available datasets with similar health status, sequencing platform and library preparation protocol to validate our results, some cells were used in more than one mixture and each dataset was split into training and testing (50%:50%), meaning that cells from one individual were present both in training and test sets but a given cell was only present in one split. Additionally, we generated scenarios where cells from a given individual were used only in one split (training or test) by assigning half of the samples to each split prior to selecting the cells based on the cell type. These led to slightly higher RMSE and lower Pearson correlation values compared to those where cells from one individual were present in both splits, but the same conclusions hold true

in both analyses (Supplementary Figures 25-26).

Blinding

Blinding is not relevant for this study, as no treatment was evaluated.

Reporting for specific materials, systems and methods

We require information from authors about some types of materials, experimental systems and methods used in many studies. Here, indicate whether each material, system or method listed is relevant to your study. If you are not sure if a list item applies to your research, read the appropriate section before selecting a response.

Materials & experimental systems

n/a	Involved in the study
☒	☐ Antibodies
☒	☐ Eukaryotic cell lines
☒	☐ Palaeontology and archaeology
☒	☐ Animals and other organisms
☒	☐ Human research participants
☒	☐ Clinical data
☒	☐ Dual use research of concern

Methods

n/a	Involved in the study
☒	☐ ChIP-seq
☒	☐ Flow cytometry
☒	☐ MRI-based neuroimaging
